# Global Patterns of Human Rhinovirus Activity and Epidemic Duration, 2016–2025: Before, During, and After the COVID-19 Pandemic

**DOI:** 10.3390/pathogens15040446

**Published:** 2026-04-20

**Authors:** Alessandra Picelli, Emma Papini, Guglielmo Bonaccorsi, Angela Bechini, Fabiola Berti, Sara Boccalini, Paolo Bonanni, Manuela Chiavarini, Claudia Cosma, Chiara Lorini, Cristina Salvati, Valentina Saviozzi, Patrizio Zanobini, Marco Del Riccio, Saverio Caini

**Affiliations:** Department of Health Sciences, University of Florence, Viale Morgagni 48, 50134 Florence, Italy

**Keywords:** human rhinovirus, seasonality, global surveillance, epidemic timing, epidemic duration

## Abstract

Background: Human rhinoviruses (HRVs) exhibit a global circulation characterized by prolonged epidemics and a less concentrated seasonal distribution compared with other respiratory viruses. In this study, we describe the timing, amplitude and duration of HRV epidemics on a global scale, analyzing seasonal patterns in relation to geographic latitude. Methods: HRV surveillance data reported to WHO FluNet from 2016 to 2025 were analyzed. Epidemic peak timing, amplitude and duration were estimated as a function of geographic latitude using harmonic analyses, with a comparison between the pre-pandemic (2016–2019) and post-pandemic (2021–2025) periods. Results: During the study period, 432,399 HRV detections were reported to WHO FluNet across 50 countries. Among these, 24 countries met the predefined criteria for seasonal analysis. Epidemic peak timing showed differences consistent with latitude, with peaks occurring in late autumn and winter in the Northern Hemisphere, during the central months of the year in the Southern Hemisphere, and greater temporal variability in the intertropical belt. Peak amplitude showed marked heterogeneity across countries (median 68.2%, range 28.1–96.7%), while epidemic duration indicated prolonged circulation (median 31 weeks, range 5–48 weeks). A secondary seasonal peak was identifiable in most countries, further supporting the relatively diffuse seasonal profile of HRV circulation. Comparison between the pre- and post-pandemic periods showed largely stable peak timing in most countries, alongside heterogeneous changes in peak amplitude. Conclusions: HRV is characterized by prolonged and weakly concentrated seasonal activity, with epidemic circulation often extending over several months. Despite major epidemiological perturbations during the COVID-19 pandemic, the timing of seasonal peaks remained largely stable across countries, highlighting the epidemiological resilience of HRV and the need for continuous, pathogen-specific surveillance.

## 1. Introduction

Acute respiratory infections represent a major global public health problem. Among respiratory viruses, human rhinoviruses (HRVs) are consistently reported as one of the main causative agents of respiratory tract infections in all age groups and in different healthcare settings, contributing substantially to outpatient visits, emergency room admissions, hospitalizations and exacerbations of chronic respiratory diseases [1,2]. Although traditionally considered responsible for mild clinical conditions limited to the upper respiratory tract, growing evidence indicates that HRV can also affect the lower respiratory tract, causing bronchiolitis, pneumonia and wheezing episodes, particularly in children, older adults, individuals with asthma and immunocompromised patients [1,3]. Consequently, the clinical and economic burden of HRV infections is considerable, making this pathogen relevant far beyond the traditional concept of the “common cold” [2,3].

HRVs belong to the *Picornaviridae* family and comprise three main species (HRV-A, HRV-B and HRV-C), characterized by high genetic heterogeneity. This marked diversity, due to the high variability of the capsid regions and the frequent mutation and recombination events typical of RNA viruses, has historically complicated the classification and epidemiological tracking of circulating strains [1,4]. Furthermore, for many years, HRV surveillance has been limited by insensitive diagnostic methods and the difficulty of culturing certain strains, particularly HRV-C, which does not grow in standard culture systems [1]. The introduction of molecular techniques based on RT-PCR represented a turning point, as it significantly improved the ability to detect infections and enabled large-scale molecular epidemiology studies [4,5]. However, the high number of co-circulating subtypes and rapid genetic turnover continue to pose a challenge for interpreting patterns of transmission, seasonality and association with clinical severity [5].

Clinical surveillance studies show that human rhinoviruses are a stable component of acute respiratory infections (ARIs) in healthcare settings. In a national surveillance system conducted in China between 2012 and 2021, HRV was detected in 7.54% of patients with laboratory-confirmed ARI, ranking among the most frequently identified respiratory viruses and being continuously detected in both outpatients and hospitalized patients [6]. The same study also documented the presence of HRV in all age groups, with a clinically relevant contribution to lower respiratory tract infections, including pneumonia. Similarly, data from European university hospitals show that HRV is frequently detected by molecular diagnostics in patients hospitalized for respiratory distress, with a significant proportion of cases associated with pneumonia [7]. A substantial proportion of these patients were also admitted to intensive care, indicating a clinically significant impact even among adult and frail populations. Overall, this evidence indicates that HRV represents a frequent finding within clinical surveillance studies of respiratory infections and contributes substantially to the clinical burden of ARIs, including severe disease requiring hospitalization.

Surveillance analyses have suggested that HRV circulation may have been less affected by the general reduction in respiratory virus circulation during the COVID-19 pandemic and that transmission resumed relatively early in the post-pandemic phase [8]. Consistently, a global multicenter study reported virus-specific differences in the timing of circulation recovery, with HRV showing an earlier resurgence compared with several other respiratory pathogens [9]. The characterization of HRV temporal dynamics remains complex, as the currently available evidence derives from heterogeneous surveillance systems with variable geographical coverage and methodological approaches. Therefore, an accurate assessment of the timing, amplitude, and duration of seasonal HRV epidemics is a fundamental step towards understanding their epidemiological dynamics and guiding more effective surveillance and prevention strategies. In this study, we aim to quantify and compare global seasonal patterns of human rhinovirus circulation using FluNet surveillance data during 2016–2025 (thus incorporating both pre- and post-COVID-19 seasons).

## 2. Methods

### 2.1. Data Sources Definitions

Surveillance data on laboratory-confirmed HRV detections were retrieved weekly from the WHO FluNet database (https://www.who.int/tools/flunet, accessed on 15 January 2026). Countries were grouped into three major latitudinal zones according to the location of their geographic centroid: Northern Hemisphere (north of the Tropic of Cancer), Southern Hemisphere (south of the Tropic of Capricorn), and the intertropical belt (ITB), defined as the area between the two tropics. Given the marked latitudinal differences in respiratory virus seasonality, the epidemiological “season” was defined using region-specific criteria. For countries located in the intertropical belt and in the Southern Hemisphere, seasons were aligned with the calendar year (weeks 01–52/53), whereas for Northern Hemisphere countries the season was defined as the period spanning from week 27 of one year to week 26 of the subsequent year, in accordance with previous methodological approaches [10]. According to this seasonal framework, up to nine complete surveillance seasons were eligible for analysis. Specifically, nine consecutive seasons (from 2016–2017 to 2024–2025) were analyzed for Northern Hemisphere countries, while ten calendar-year seasons (from 2016 to 2025) were considered for countries in the intertropical belt and Southern Hemisphere. To avoid the inclusion of incomplete seasonal periods, data from weeks 1–26 of 2016 and from week 27/2025 onwards were excluded for Northern Hemisphere countries, while weeks 1–3 of 2026 were excluded for countries in the intertropical belt and Southern Hemisphere. The unit of analysis was defined as the “country-season”, corresponding to the set of weekly surveillance data collected for a given country within a single epidemiological season.

In the FluNet database, country-level surveillance data are classified into three categories according to the data source: (i) sentinel surveillance, referring to information routinely collected through structured sentinel systems; (ii) non-sentinel surveillance, including data derived from outbreak investigations, universal or point-of-care testing, or other sources outside sentinel surveillance; (iii) not defined, comprising records without a specified surveillance framework, which may reflect a combination of sentinel and non-sentinel contributions. For some country-season combinations, data were available from more than one surveillance source. Since the timing and duration of HRV circulation are not expected to differ according to the clinical severity of detected cases, a single dataset was selected for each country-season, retaining the one with the highest number of HRV detections in order to maximize statistical power and improve the robustness of peak timing estimates. Prior to this selection step, country-seasons with fewer than 30 reporting weeks were excluded, regardless of surveillance category or total number of detections. This criterion was applied to minimize incomplete seasonal coverage and to reduce potential bias in the estimation of epidemic onset, peak timing, and overall epidemic duration. FluNet does not report the total number of specimens tested for HRV; therefore, virus-specific positivity rates could not be calculated. Consequently, the analysis was restricted to the investigation of seasonal circulation patterns, with particular emphasis on the temporal position of epidemic peaks and on the length of epidemic activity periods.

### 2.2. Statistical Analysis

For each country-season, the total number of HRV detections reported to FluNet was calculated and grouped into three categories (1–24, 25–49 and ≥50 detections). Country-seasons with zero reported detections were excluded, as the absence of virus circulation could not be distinguished from a lack of testing activity. The distribution of detection categories was subsequently summarized by latitudinal zone, WHO region and season. Seasonal patterns of HRV circulation were investigated using country-specific time series analyses performed with the EPIPOI software (version 2017/10; https://www.epipoi.info/, accessed on 3 February 2026). The 2020 season (2020–2021 for Northern Hemisphere countries) was retained in the overall analyses, given the sustained circulation of HRV during the COVID-19 pandemic period. However, for comparative analyses of pre-pandemic and post-pandemic circulation patterns, the 2020 season was excluded in order to minimize potential transitional effects related to the early pandemic phase.

The main objective was to describe global HRV epidemic dynamics and to compare circulation patterns before and after the COVID-19 pandemic period. To improve the robustness of the results, only countries reporting at least 50 HRV detections in a minimum of three seasons, either in the pre-pandemic or post-pandemic period (or both), were retained for the final analysis.

Within EPIPOI, each time series is first detrended by fitting a quadratic polynomial, after which the periodic annual function (PAF) is reconstructed using Fourier decomposition including annual, semi-annual and quarterly harmonic components. In interpreting the results of this analysis, it should be kept in mind that the PAF represents a simplified description of the main seasonal pattern and may therefore not fully capture multiple peaks or short-term fluctuations. The timing of the epidemic peak is defined as the calendar month corresponding to the maximum value of the PAF, while peak amplitude is calculated as the ratio between the wave height and the peak value and expressed as a percentage. This metric reflects the concentration of detections around the main seasonal peak and, by definition, may occasionally exceed 100%. In addition to the primary seasonal peak, we recorded the timing and amplitude of a potential secondary peak when a second distinct peak was identifiable within the same annual cycle in the PAF. Peak timing and amplitude were initially estimated at country level using data from the entire observation period (2016–2025) and subsequently recalculated separately for the pre-pandemic (2016–2019) and post-pandemic (2021–2025) periods for countries meeting the predefined data availability criteria. Epidemic duration was estimated for each country-season using the 75% Average Annual Percentage (AAP) approach, which identifies the shortest sequence of consecutive weeks accounting for at least 75% of the total annual number of detections [10,11,12,13].

### 2.3. Software

All statistical analyses were conducted using Stata version 17 (StataCorp, College Station, TX, USA) and the open-access EPIPOI software (version 2017/10; https://www.epipoi.info/, accessed on 3 February 2026).

## 3. Results

### 3.1. Data Availability and Descriptive Summary

**Figure 1:**
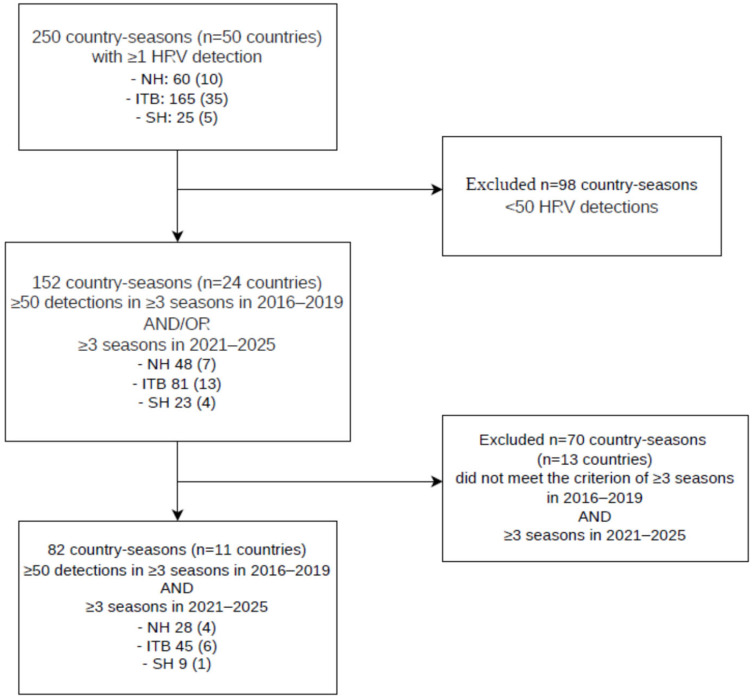
Flow diagram of country selection for the analysis of human rhinovirus (HRV) seasonal circulation using WHO FluNet data (2016–2025), showing inclusion and exclusion steps for the overall seasonal analyses and for the pre- and post-pandemic comparison.

Over the study period, 432,399 HRV detections were reported to FluNet from 50 countries across different geographical settings, including the Northern Hemisphere (n = 10), the intertropical belt (n = 35) and the Southern Hemisphere (n = 5). Figure 1 summarizes the country selection process and inclusion criteria for the seasonal analyses and the pre- and post-pandemic comparison. The distribution of reports showed high variability: the median number of detections per season was 171, with approximately one fifth of seasons (20.8%, n = 52) characterized by a low number of reports (<25), while more than two thirds (70.4%, n = 176) exceeded the threshold of 50 detections. Details on the distribution by latitudinal area, WHO region and season are reported in Supplementary Tables S1–S3.

Substantial differences in the number of HRV detections emerged across latitude-defined areas. In particular, the median number of seasonal detections was 89 in countries in the intertropical belt, 382 in the Northern Hemisphere, and 1924 in the Southern Hemisphere (Supplementary Table S1). In analyses stratified by WHO region (Supplementary Table S2), the largest share of overall detections was from the Region of the Americas (324,215 detections; median = 132 per season), followed by the Western Pacific Region (68,975; median = 365) and the Eastern Mediterranean Region (30,389; median = 331). In contrast, lower numbers of reported HRV detections were observed in the African Region and South-East Asia, while no reports were recorded from the WHO European Region in the FluNet dataset for HRV during the study period (despite the existence of well-established respiratory virus surveillance systems in several European countries).

When considering temporal trends, annual reports showed marked variability (Supplementary Table S3). After the minimum observed in 2019, with 10,461 reported detections, a progressive increase was documented, with a peak in 2024 (110,650 detections). In parallel, the seasonal median also increased in the most recent period, rising from 70 in 2019 to 374 in 2025.

### 3.2. Peak Timing and Duration of HRV Epidemics

Based on the cut-offs described in the Section 2, the primary seasonal peak and its main characteristics (peak month, amplitude, and duration) were identified for 24 of the 50 countries with at least one reported detection during the study period (the completed time-series are available as Supplementary Figures S1–S34). The median epidemic duration at country level was 31 weeks, with an overall range from 5 to 48 weeks (Table 1). The amplitude of the primary peak showed marked between-country variability, with a global median of 68.2% and values ranging from 28.1% in Colombia to 96.7% in Uruguay.

**Figure 2:**
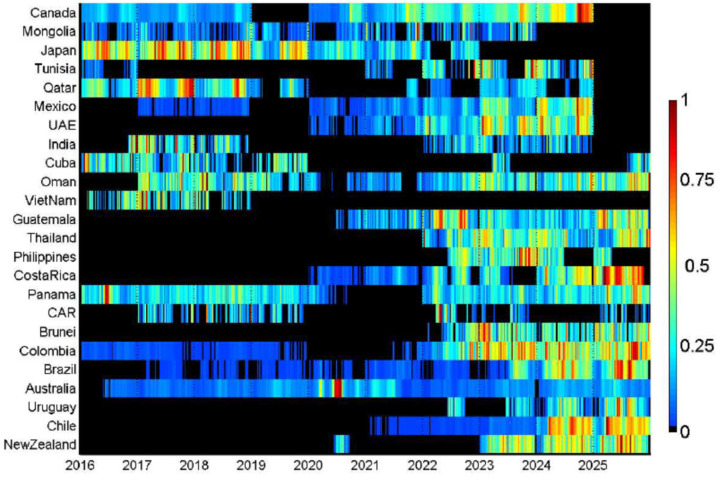
Heatmap of HRV detections by country and month. Colour gradients indicate the relative intensity of monthly detections for each country and time period, ranging from low values (blue) to high values (red).

**Figure 3:**
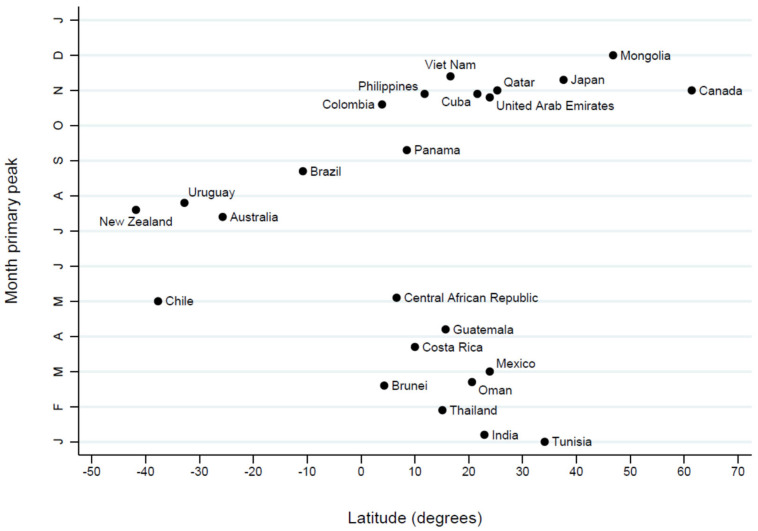
Timing of the primary seasonal HRV peak across countries by latitude in the global dataset, 2016–2025.

A secondary seasonal peak was identifiable in most countries included in the analysis, being present in 21 of the 24 countries evaluated. Among countries with a detectable secondary peak, the secondary peak occurred several months apart from the primary peak in most cases. The amplitude of the secondary peak ranged between 13.5% and 80.3% (median: 38.5%). No secondary peak was identified in Uruguay, Brazil, and Tunisia (Table 1). Both primary peak timing and amplitude displayed clear latitudinal structuring, although several countries did not fully conform to the prevailing hemispheric patterns. In the Southern Hemisphere, peak activity was concentrated around the middle of the year, with Chile peaking in May and Australia, Uruguay and New Zealand showing maxima mainly between July and August. In the Northern Hemisphere, peak timing differed according to latitude: countries located at higher latitudes experienced peaks typically in late autumn or early winter (Canada and Mongolia in November–December, Japan in November), whereas lower-latitude Northern countries tended to peak earlier from early winter to early spring (Tunisia in January; Thailand in February; India in January–February; Mexico and Oman in March; Guatemala and Costa Rica in April–May). Within the intertropical belt, peak timing was less concentrated within a single seasonal window and was predominantly observed either between February and May or between October and November with a few exceptions (Figure 2 and Figure 3). As shown in Table 2, the timing of the primary rhinovirus seasonal peak appeared comparable between the pre-COVID period (2016–2019) and the post-COVID period (2021–2025) across most countries. Changes in peak timing were observed in Panama (from a summer peak to a spring peak), Oman (from a spring peak to a winter peak) and Mexico (from a spring peak to an autumn peak).

**Figure 4:**
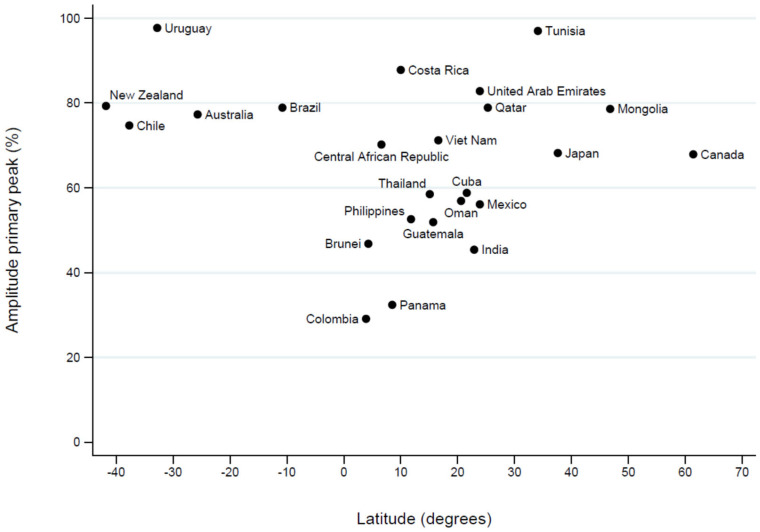
Association between country latitude and the amplitude of the primary HRV seasonal peak.

The amplitude of the primary rhinovirus seasonal peak also showed marked heterogeneity across countries (Table 1 and Figure 4). Lower values, reflecting a less pronounced seasonal peak relative to the annual baseline, were observed in tropical countries such as Colombia, whereas higher values were observed in temperate countries of the Southern Hemisphere (Australia, Chile and New Zealand), with Uruguay showing the highest peak amplitude, as well as in temperate countries of the Northern Hemisphere such as Tunisia.

Overall, rhinovirus epidemics were characterized by long and variable durations, as estimated using the 75% AAP method, with epidemic activity often extending over several months within a given season (Table 1). When comparing the pre-pandemic (2016–2019) and post-pandemic (2021–2025) periods, the amplitude of the primary seasonal peak showed heterogeneous changes across countries, with no uniform pattern. In some countries, a reduction in peak amplitude was observed after 2020, whereas in other values remained broadly stable or increased (Table 2).

## 4. Discussion

In this study, we provide a comprehensive characterization of the seasonal circulation patterns of HRV at a global scale, based on analyses of long-term surveillance data from WHO FluNet. Respiratory viruses exhibit heterogeneous seasonal behavior across countries and latitudes, and this variability has been suggested to be influenced by a combination of climatic conditions and latitude-related environmental factors that modulate virus stability, transmission dynamics and host susceptibility [14]. This aspect is particularly relevant when comparing different respiratory viruses, as similar climatic and environmental drivers may shape seasonal circulation while giving rise to markedly different epidemiological patterns. Such differences may reflect virus-specific characteristics, including variations in environmental stability and transmissibility under different temperature and humidity conditions, as well as the contribution of additional biological mechanisms, such as virus–virus interactions. These differences in circulation patterns are likely supported by HRV-specific biological characteristics: high genetic and antigenic diversity, marked genomic heterogeneity among circulating strains, and frequent recombination events limit the development of cross-protective immune responses and promote repeated reinfections over time [15]. In this context, HRV displays more temporally diffuse and less sharply defined seasonal patterns than other major respiratory pathogens, such as influenza viruses. Moreover, HRV shows relatively continuous circulation and asynchronous temporal dynamics compared with influenza, possibly related to virus-virus interaction phenomena [16]. Given the ecological and detection-based nature of the data, these interpretations should be considered hypothesis-generating rather than causal.

In line with evidence indicating a limited reduction in HRV circulation during the pandemic and its early subsequent resurgence [8,9], our FluNet-based analysis suggests that HRV maintained sustained circulation during the pandemic phase and rapidly re-established well-defined seasonal patterns in the following years. The increase in HRV detections observed after 2020 may partly reflect changes in surveillance intensity, diagnostic capacity, and testing practices during the COVID-19 pandemic, in addition to possible true epidemiological changes [17]. As this study is based on aggregated surveillance data, it is not possible to disentangle these factors or to infer causality. This behavior differs from that observed for other major respiratory viruses, which exhibited more prolonged suppression and delayed recovery of circulation [9,18]. In addition, PCR-based evidence has shown that HRV is frequently detected in asymptomatic individuals, suggesting that subclinical and pauci-symptomatic infections may contribute substantially to the maintenance of viral circulation in the population [19]. Overall, these factors contribute to the relative epidemiological resilience of HRV and reinforce the need for continuous, pathogen-specific surveillance strategies.

Our results highlight a clear latitudinal structuring of HRV seasonal activity, with circulation peaks predominantly concentrated in late autumn and winter in the Northern Hemisphere, during the central months of the year in the Southern Hemisphere, and with a broader temporal distribution in intertropical regions. This spatial organization is consistent with global patterns described for respiratory viruses, according to which seasonality tends to become more pronounced with increasing distance from the equator and less well defined in tropical areas [20]. In terms of epidemic duration, HRV epidemics were characterized by prolonged periods of activity and by a relatively low temporal concentration of circulation, with durations frequently extending over several months. Compared with influenza viruses, which in temperate regions typically exhibit shorter and more temporally concentrated epidemic windows [13], HRV shows a more continuous transmission profile, with substantial activity detectable even outside the core winter period [20]. In line with this continuous transmission profile, in temperate regions this results in extended activity around the seasonal peak, whereas in intertropical areas circulation remains more diffuse and less temporally predictable throughout the year.

In this context, the identification of a secondary seasonal peak in most countries further supports this pattern of extended and less concentrated circulation. Not infrequently, the secondary peak showed an amplitude only moderately lower than the primary one, suggesting that HRV activity tends to be distributed across multiple periods within the same annual cycle rather than being confined to a single dominant epidemic wave.

Against this background, the results of the present study provide additional elements to contextualize the impact of the COVID-19 pandemic on the seasonality of respiratory viruses at a global scale. In particular, despite country-specific heterogeneity, the timing of the HRV seasonal peak did not show a systematic shift between the pre-pandemic and post-pandemic periods in most of the countries analysed. This behaviour appears distinct from that reported for other respiratory viruses during the same period. For respiratory syncytial virus (RSV), off-season epidemics and substantial alterations in epidemic timing have been documented in several geographical settings following the relaxation of containment measures [9]. Comparable evidence has also been reported for human metapneumovirus and human adenovirus, based on global surveillance analyses documenting marked changes in seasonal patterns before and after the pandemic [21,22]. Against this background, the temporal displacement observed for HRV in our study appears more limited in absolute terms and largely confined to specific countries, suggesting a greater temporal continuity of its circulation. This comparison should be interpreted with caution, given differences in surveillance systems, testing strategies and analytical approaches across viruses and geographical contexts, but it contributes to strengthening the interpretation of our findings from a comparative perspective.

In light of this epidemiological context and considering the well-established evidence that HRV is associated with a non-negligible clinical burden, particularly among vulnerable populations and in hospital settings [2,3,7], our findings have direct public health relevance. In this context, the integration of HRV into respiratory virus surveillance systems appears essential to optimize healthcare service planning during periods of increased viral circulation, facilitate the early detection of changes in circulation patterns, and strengthen strategies aimed at protecting populations at higher risk. Furthermore, the geographic and latitudinal heterogeneity observed in the present study suggests that surveillance strategies and healthcare planning should be tailored to different epidemiological contexts. In temperate countries, the greater seasonal concentration of HRV during winter months supports the systematic inclusion of HRV in seasonal respiratory virus surveillance, alongside other pathogens of interest. Conversely, in intertropical regions, where circulation appears more diffuse and less seasonally defined, our findings indicate the need for continuous year-round monitoring to capture relevant temporal variations and to reduce the risk of underestimating the associated disease burden.

An important strength of this study lies in the extensive temporal coverage of the surveillance dataset, which allowed the inclusion of multiple seasons up to 2025, enabling a robust assessment of HRV circulation dynamics. In addition, the use of a standardized harmonic framework based on periodic annual functions ensured a uniform identification of epidemic timing, peak amplitude, and duration across heterogeneous geographical settings [23]. The global scope of the analysis and the application of a homogeneous analytical approach across countries improve the comparability of the results and reduce methodological heterogeneity. Overall, these features strengthen the interpretative robustness of the observed latitudinal and temporal patterns.

Despite these strengths, several limitations should be considered. First, the exclusion of seasons with a low number of detections resulted in the underrepresentation of some geographical areas, particularly in countries characterized by less structured surveillance systems. This selection process may have contributed to concentrating the analytical sample in countries with higher surveillance capacity, which should be considered when interpreting the observed latitudinal patterns. In this context, part of the apparent geographic structuring may reflect differences in data availability and reporting intensity rather than purely underlying epidemiological mechanisms. Given the heterogeneity of surveillance systems contributing to FluNet, including differences in geographical coverage, diagnostic capacity, and reporting practices, the completeness and representativeness of the data may vary both across and within countries. In large countries, surveillance data may be disproportionately derived from areas with more developed laboratory infrastructure, while more remote or resource-limited regions may be underrepresented. These inclusion criteria, while necessary to ensure sufficient temporal completeness and robustness of seasonal estimates, may preferentially retain countries with more structured surveillance systems and consistent reporting practices, potentially introducing selection bias.

In addition, differences in surveillance categories across country-seasons may have affected comparability over time within the same country. The different surveillance categories (sentinel, non-sentinel, and not defined) may reflect distinct underlying sampling frameworks; therefore, selecting the dataset with the highest number of detections may introduce some heterogeneity across country-seasons. This limitation is not specific to HRV but has also been observed in previous global analyses of other respiratory viruses, including human adenovirus and human metapneumovirus, based on FluNet data [21,22]. This finding once again highlights the need to strengthen and expand virological surveillance systems at a global scale. This is especially important given that several underrepresented regions correspond to highly populated countries, where limited surveillance coverage may hinder the timely detection of changes in circulation patterns as well as the emergence and spread of novel viral strains.

Second, the lack of denominators in the FluNet database prevented the calculation of HRV-specific positivity rates, restricting the analysis to the assessment of relative temporal patterns rather than absolute incidence estimates. This assessment is based on the characteristics of the FluNet dataset analyzed in this study, including the number of reporting countries, the number of available reporting weeks per season, and the overall distribution of HRV detections across regions. These differences likely reflect not only true epidemiological variability but also heterogeneity in surveillance capacity, laboratory infrastructure, and reporting systems across countries. Within-country variability may further contribute to these patterns, particularly in large countries where surveillance systems may not uniformly cover all geographical areas.

Differences in testing strategies, laboratory capacity, and reporting practices may also have influenced detection intensity. In addition, FluNet data primarily reflect laboratory-confirmed cases identified within healthcare settings and may therefore underestimate mild or community-managed infections, which are particularly frequent in the case of HRV. HRV detection can occur in asymptomatic individuals and may reflect prolonged viral shedding, which can complicate the interpretation of PCR-based detections as indicators of incident symptomatic infections. Epidemic duration estimates based on the 75% AAP method may be influenced by the choice of threshold, particularly for viruses such as HRV characterized by prolonged and less concentrated circulation, and different thresholds may yield different duration estimates. Despite these limitations, the consistency of the seasonal patterns observed across different geographical contexts supports the overall robustness of the main findings. Alternative empirical approaches to peak identification may yield slightly different estimates compared to those derived from the harmonic model. Finally, peak timing and epidemic duration were estimated as point estimates without formal uncertainty quantification, and this should be considered when interpreting small differences, particularly in pre- and post-pandemic comparisons.

In conclusion, our findings identify HRV as a respiratory pathogen characterized by marked epidemiological resilience, reflected in its sustained circulation, a broad seasonal profile, and the ability to rapidly re-establish coherent seasonal patterns following major epidemiological perturbations. The global and harmonized analysis presented in this study underscores the epidemiological relevance of HRV and complements existing evidence indicating a non-negligible public health impact. From a public health perspective, these findings support the systematic integration of HRV into year-round respiratory virus surveillance systems and reinforce the value of continuous monitoring to improve the understanding of viral circulation dynamics and to inform more effective management of healthcare resources and protection strategies for vulnerable populations.

## Figures and Tables

**Table 1:** Month, amplitude, and duration of the primary and secondary seasonal HRV peak by country (2016–2025).

		2016–2025					
Country	Latitude	N. Season with ≥50 Reported Rhinovirus Detections	Duration in Weeks (75% AAP), Range (Median)	Month Primary Peak	Amplitude Primary Peak (%)	Month Secondary Peak	Amplitude Secondary Peak (%)
New Zealand	−41.8	4 (2020, 2023–2025)	7 to 33 (32)	6.6 (Jul)	78.3	3.0 (Mar–Apr)	53.3
Chile	−37.7	5 (2021–2025)	23 to 34 (33)	4.0 (Apr–May)	73.7	8.1 (Sep)	48.4
Uruguay	−32.8	4 (2022–2025)	10 to 26 (20)	6.8 (Jul)	96.7	-	-
Australia	−25.7	10 (2016–2025)	22 to 36 (32)	6.4 (Jul)	76.3	2.9 (Mar)	33.2
Brazil	−10.8	9 (2017–2025)	14 to 32 (28)	7.7 (Aug)	77.9	-	-
Colombia	3.9	9 (2016–2019, 2021–2025)	14 to 38 (30)	9.6 (Oct)	28.1	4.6 (May)	15.2
Brunei Darussalam	4.3	4 (2022–2025)	20 to 41 (35)	1.6 (Feb)	45.8	10.8 (Nov)	29.0
Central African Republic	6.6	6 (2017–2019, 2022–2023, 2025)	12 to 40 (25)	4.1 (May)	69.2	11.3 (Dec)	13.5
Panama	8.5	9 (2016–2020, 2021–2025)	12 to 37 (34)	8.3 (Sep)	31.4	4.3 (May)	28.7
Costa Rica	10.0	6 (2020–2025)	13 to 35 (29)	2.7 (Mar)	86.8	9.1 (Oct)	67.7
Philippines	11.8	4 (2022–2025)	12 to 34 (20)	9.9 (Oct)	51.6	0.5 (Jan)	42.1
Thailand	15.1	4 (2022–2025)	28 to 38 (30)	0.9 (Jan)	57.5	6.5 (Jul)	48.7
Guatemala	15.7	6 (2020–2025)	16 to 39 (36)	3.2 (Apr)	50.9	9.2 (Oct)	35.6
Viet Nam	16.6	3 (2016–2018)	27 to 42 (36)	10.4 (Nov)	70.2	1.5 (Feb)	61.2
Oman	20.6	9 (2017–2024)	28 to 48 (36)	1.7 (Feb)	55.9	9.2 (Oct)	39.8
Cuba	21.6	6 (2016–2019, 2023, 2025)	10 to 40 (26)	9.9 (Oct)	57.8	2.1 (Mar)	37.0
India	22.9	6 (2016–2018, 2022–2024)	5 to 40 (28)	0.2 (Jan)	44.4	7.9 (Aug)	21.6
United Arab Emirates	23.9	5 (2020–2024)	28 to 34 (32)	9.8 (Oct)	81.8	1.5 (Feb)	80.3
Mexico	23.9	7 (2016–2017, 2019–2023)	24 to 40 (33)	2.0 (Feb–Mar)	55.1	9.9 (Oct)	45.9
Qatar	25.3	8 (2016–2019, 2021–2024)	7 to 37 (25)	10.0 (Oct–Nov)	77.9	3.9 (Apr)	38.5
Tunisia	34.1	5 (2016, 2021–2024)	18 to 23 (21)	12.0 (Dec–Jan)	96.0	-	-
Japan	37.6	7 (2016–2022)	23 to 42 (38)	10.3 (Nov)	67.2	6.0 (Jun–Jul)	47.2
Mongolia	46.8	8 (2016–2023)	17 to 36 (25)	11.0 (Nov–Dec)	77.6	3.4 (Apr)	18.4
Canada	61.4	8 (2016–2018, 2020–2024)	25 to 38 (36)	10.0 (Oct–Nov)	66.9	5.0 (May–Jun)	27.2

**Table 2:** Pre- and post-COVID comparison of HRV epidemic timing, duration and amplitude in selected countries (2016–2019 vs. 2021–2025).

		2016–2019			2021–2025		
Country	Latitude	Duration in Weeks (75% AAP), Range	Month Primary Peak	Amplitude Primary Peak (%)	Duration in Weeks (75% AAP), Range	Month Primary Peak	Amplitude Primary Peak (%)
Australia	−25.7	22 to 35	6.0 (Jun–Jul)	61.2	22 to 36	5.6 (Jun)	65.6
Brazil	−10.8	15 to 30	7.9 (Aug)	97.1	14 to 32	7.7 (Aug)	80.1
Colombia	3.9	28 to 37	9.2 (Oct)	47.4	14 to 38	9.6 (Oct)	24.0
Central African Republic	6.6	32 to 40	3.4 (Apr)	43.9	12 to 19	4.2 (May)	98.1
Panama	8.5	31 to 37	6.2 (Jul)	48.8	34 to 37	3.6 (Apr)	50.3
Oman	20.6	36 to 39	2.1 (Mar)	80.2	28 to 39	0.5 (Jan)	38.7
India	22.9	5 to 39	0.4 (Jan)	65.0	28 to 40	0.9 (Jan)	28.7
Mexico	23.9	32 to 36	2.1 (Mar)	84.9	29 to 40	9.9 (Oct)	55.9
Qatar	25.3	21 to 37	9.7 (Oct)	76.4	7 to 32	10.4 (Nov)	98.3
Mongolia	46.8	19 to 28	11.9 (Dec)	90.2	18 to 36	10.7 (Nov)	86.5
Canada	61.4	36 to 37	10.2 (Nov)	69.7	35 to 38	10.0 (Oct–Nov)	67.6

## Data Availability

The data supporting the findings of this study are publicly available and can be downloaded from the WHO FluNet website. The original contributions presented in this study are included in the article/Supplementary Materials. Further inquiries can be directed to the corresponding author.

## Supplementary Materials

The following supporting information can be downloaded at: https://www.mdpi.com/article/10.3390/pathogens15040446/s1, Figures S1–S34: Time-series of rhinovirus circulation by country. WHO FluNet, 2016–2025; Table S1: Global circulation of rhinovirus in countries lying in the Northern hemisphere, inter-tropical belt, or Southern hemisphere. WHO FluNet, 2016–2025; Table S2: Global circulation of rhinovirus in countries belonging to the different WHO regions. WHO FluNet, 2016–2025; Table S3: Global circulation of rhinovirus by season. WHO FluNet, 2016–2025.
