# Supplementary material for: Global Patterns of Human Rhinovirus Activity and Epidemic Duration, 2016–2025: Before, During, and After the COVID-19 Pandemic
Source: Pathogens. 2026 Apr 20;15(4):446. doi: 10.3390/pathogens15040446 (PMC13119256; doi:10.3390/pathogens15040446)

Supplementary Figure S1: Time-series of rhinovirus circulation in Australia. WHO FluNet, 2016-2025.

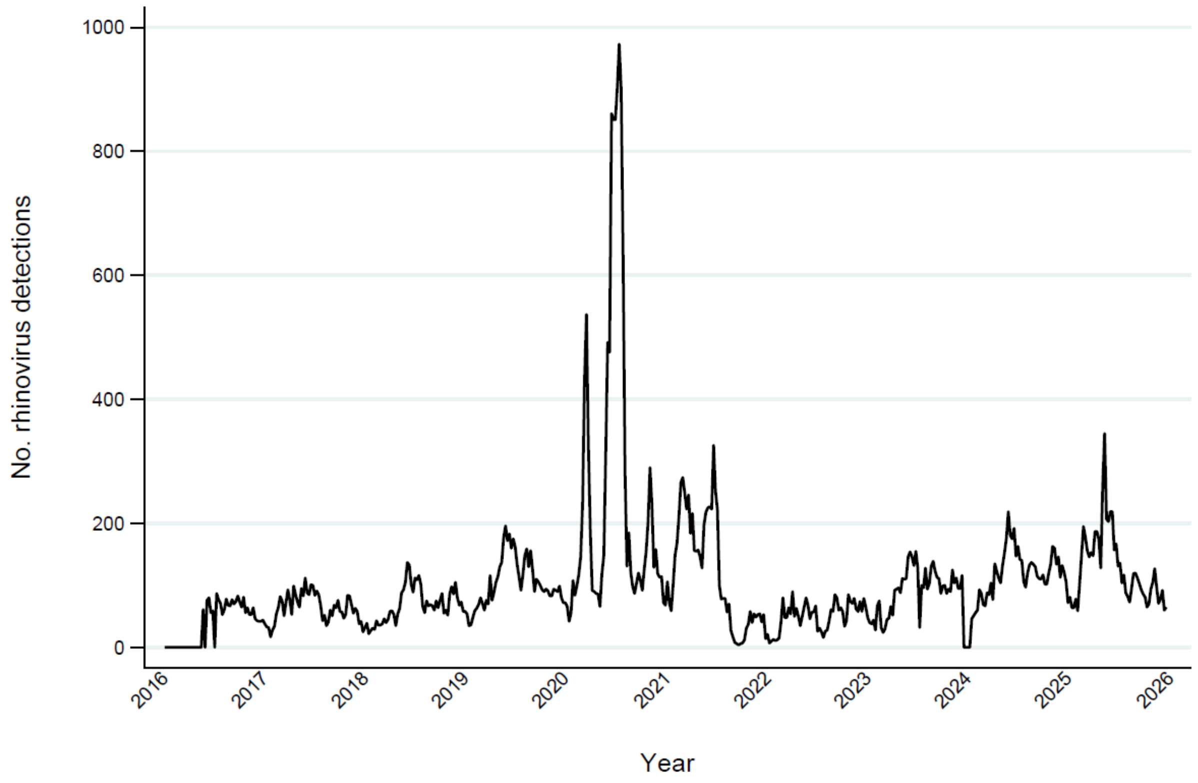

Supplementary Figure S2: Time-series of rhinovirus circulation in Barbados. WHO FluNet, 2016-2025.

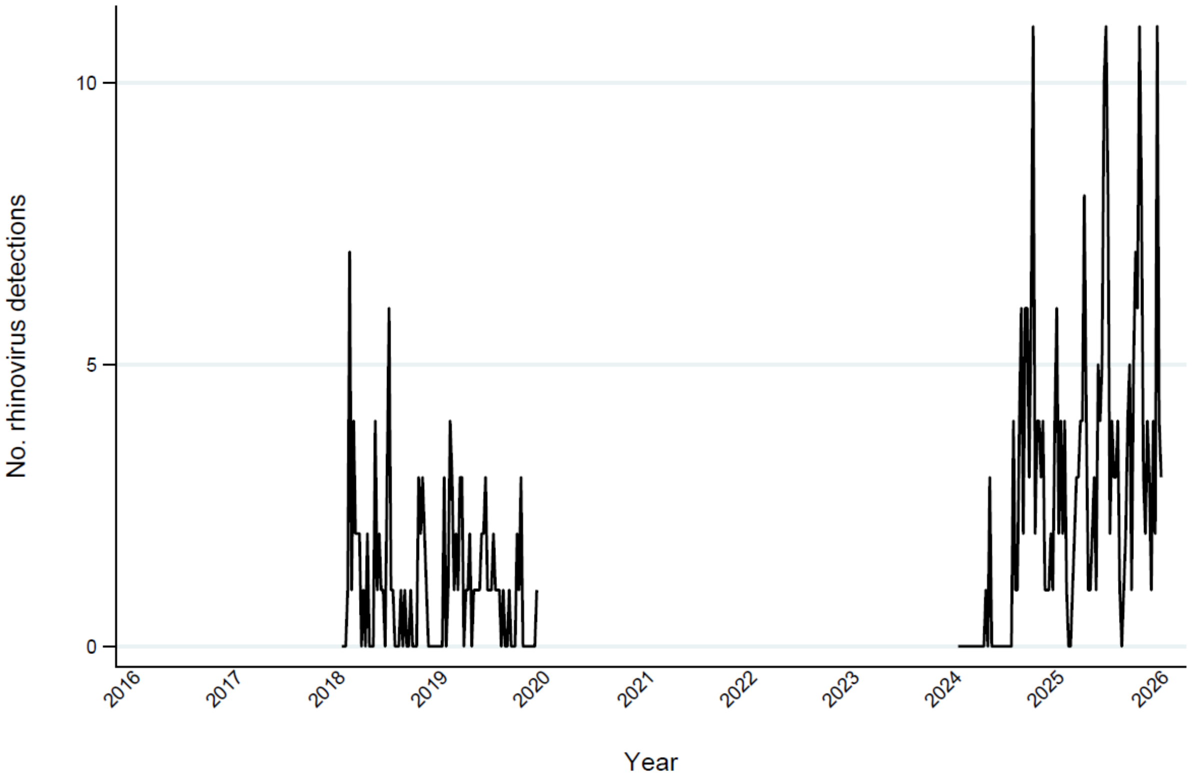

Supplementary Figure S3: Time-series of rhinovirus circulation in Belize. WHO FluNet, 2016-2025.

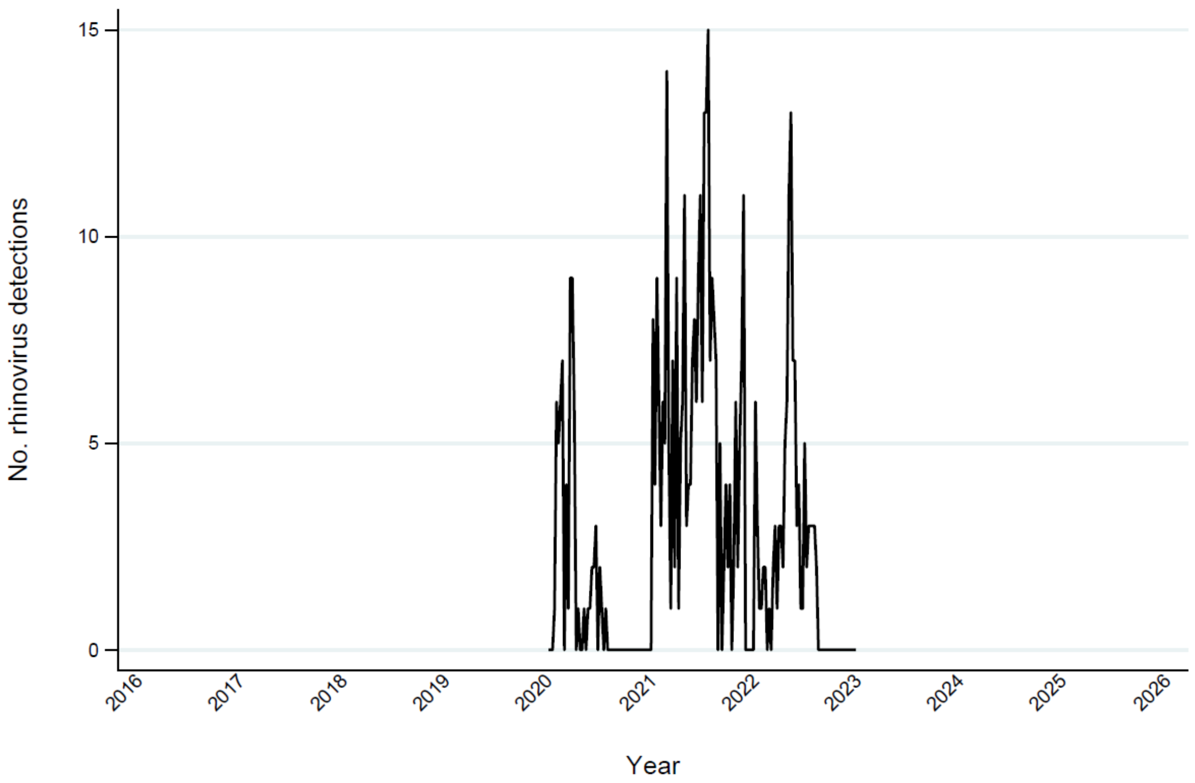

Supplementary Figure S4: Time-series of rhinovirus circulation in Bolivia. WHO FluNet, 2016-2025.

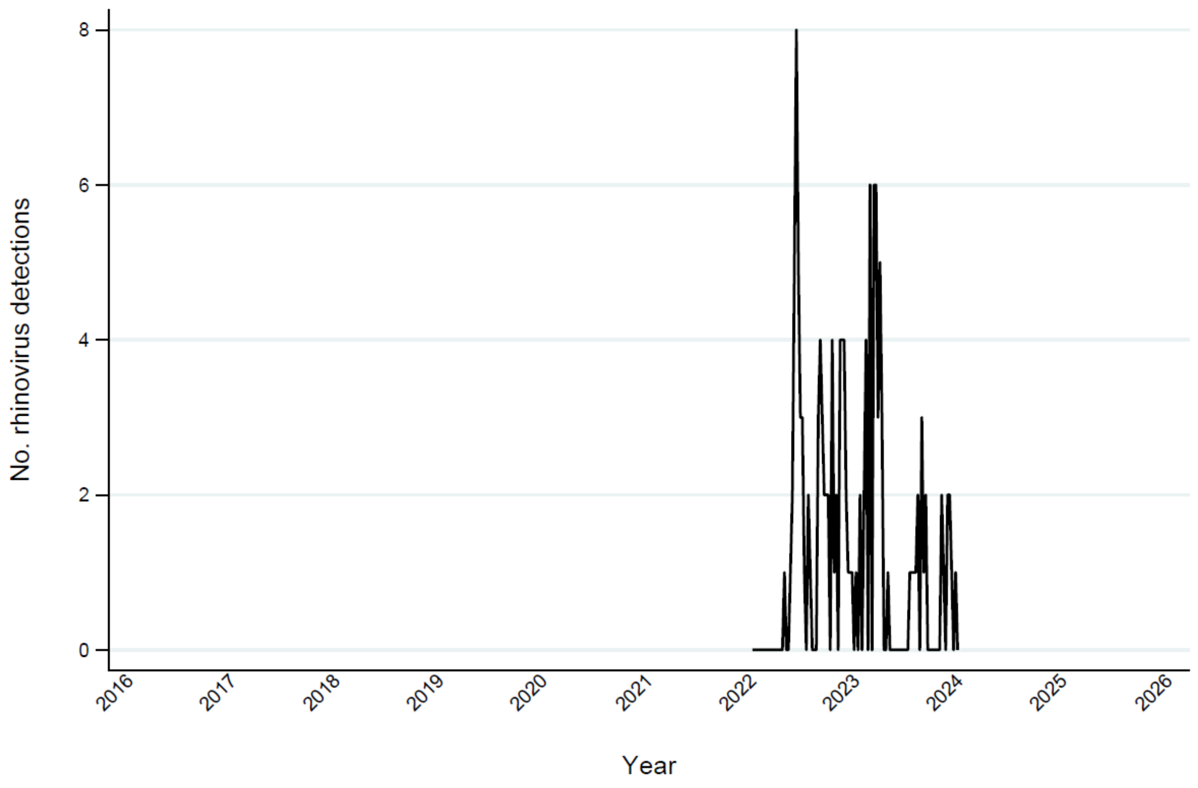

Supplementary Figure S5: Time-series of rhinovirus circulation in Brazil. WHO FluNet, 2016-2025.

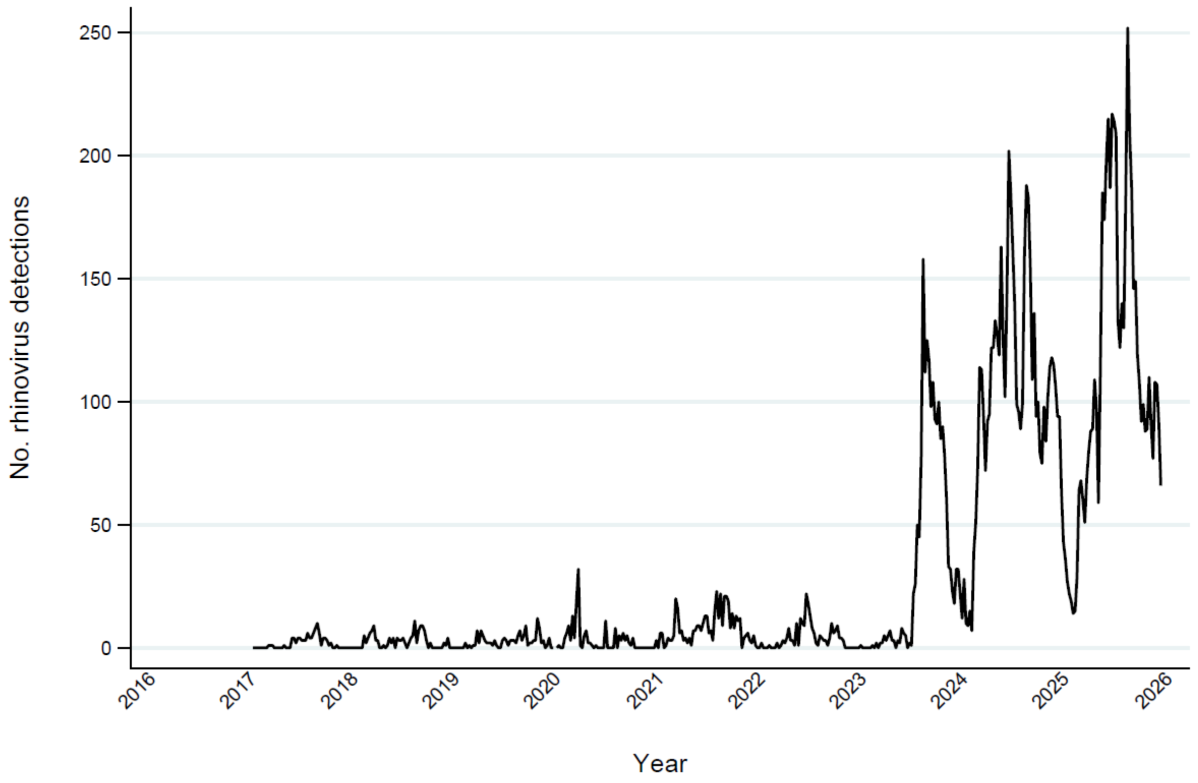

Supplementary Figure S6: Time-series of rhinovirus circulation in Brunei Darussalam. WHO FluNet, 2016-2025.

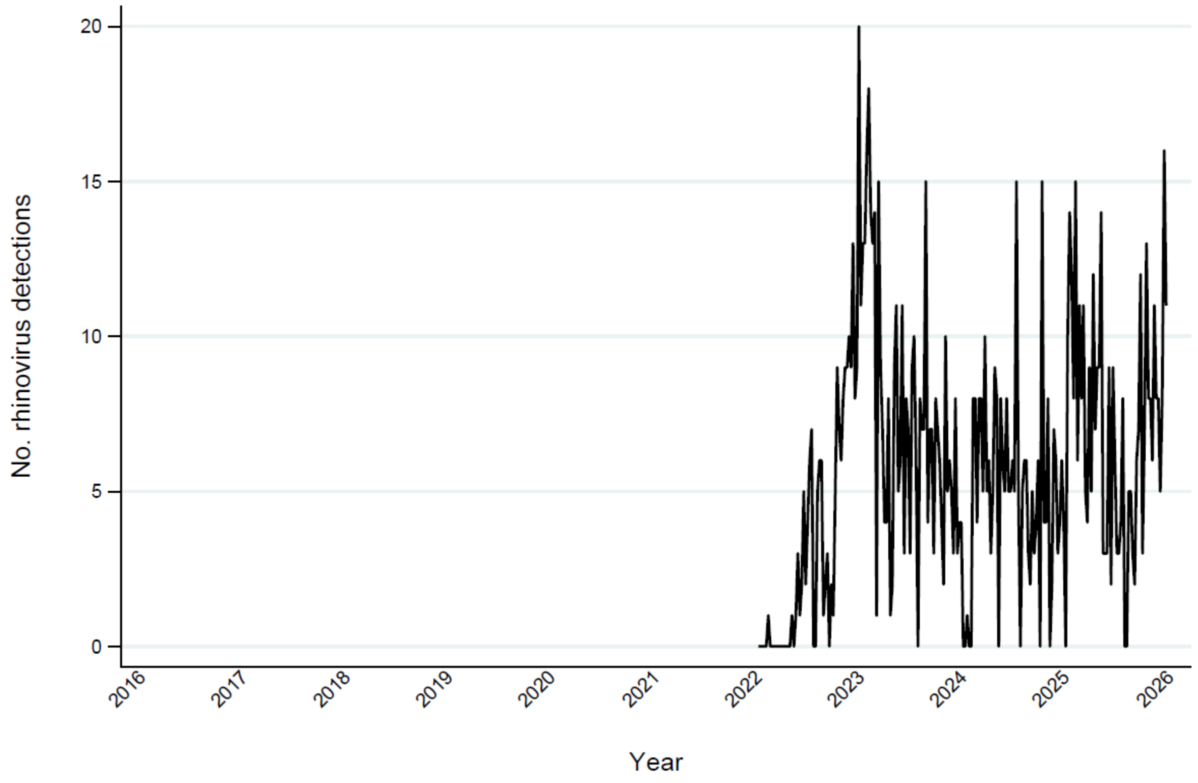

Supplementary Figure S7: Time-series of rhinovirus circulation in Canada. WHO FluNet, 2016-2025.

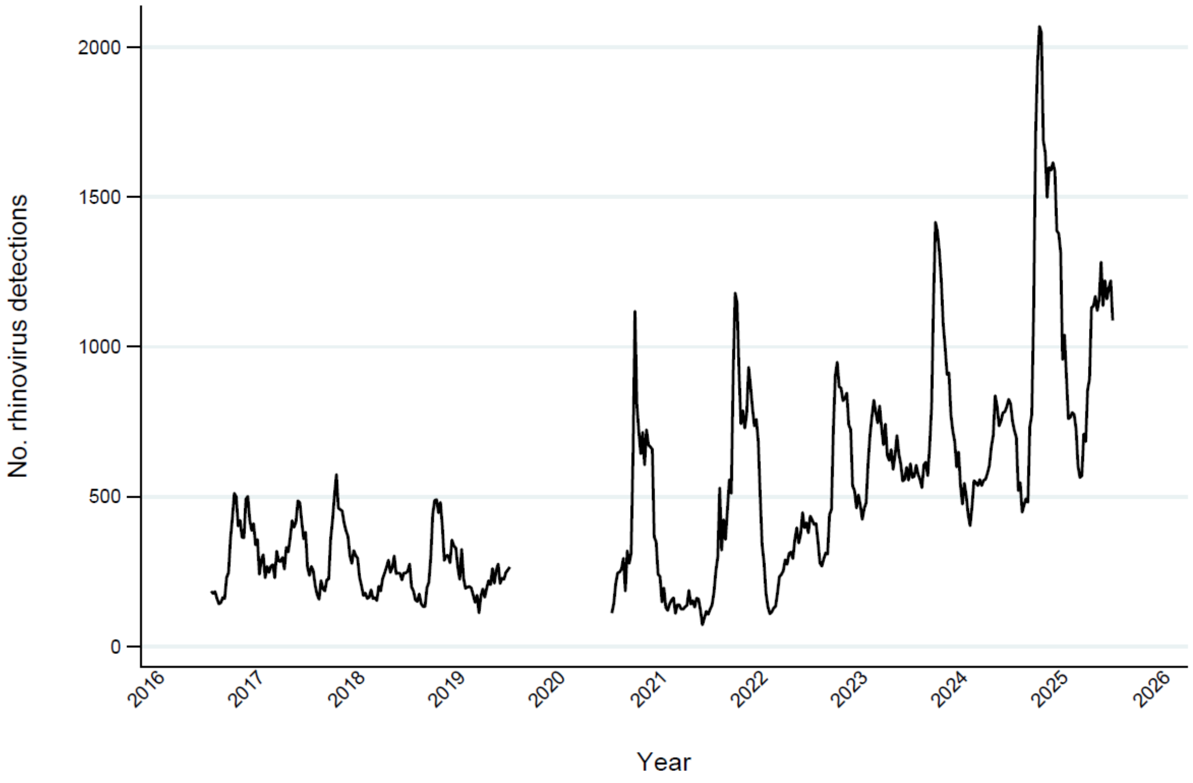

Supplementary Figure S8: Time-series of rhinovirus circulation in the Central Africa Republic. WHO FluNet, 2016-2025.

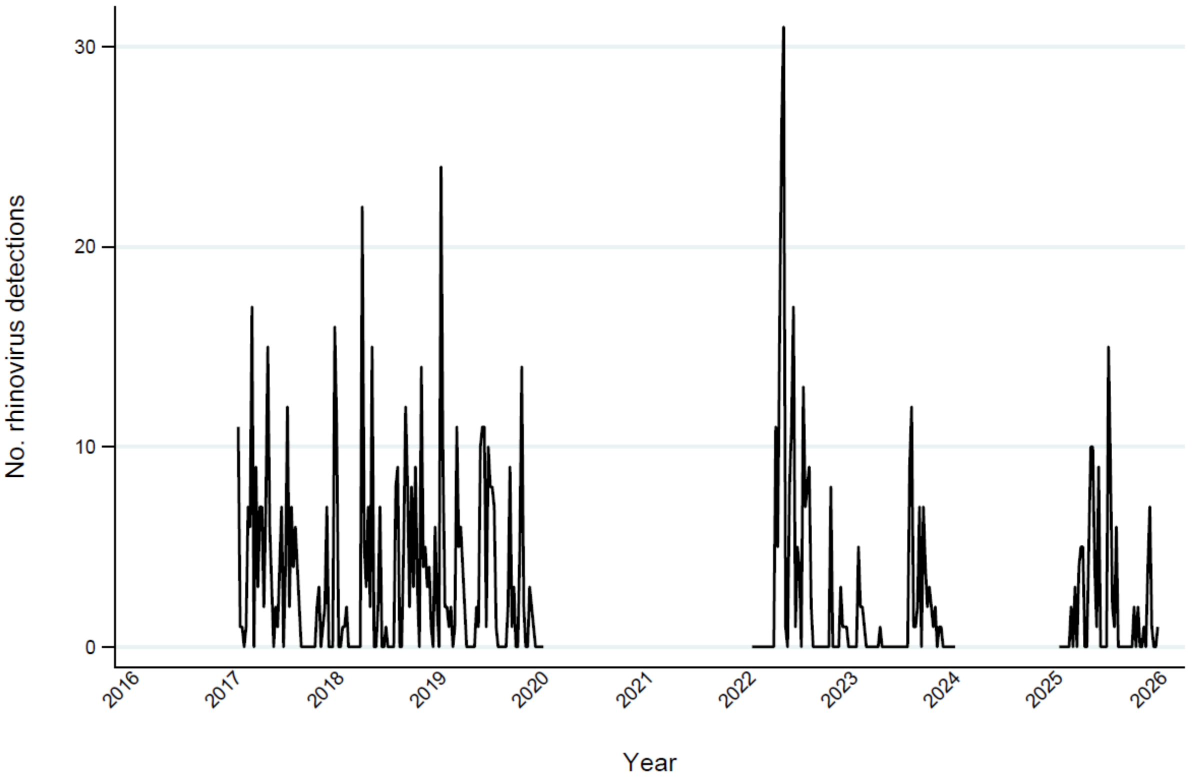

Supplementary Figure S9: Time-series of rhinovirus circulation in Chile. WHO FluNet, 2016-2025.

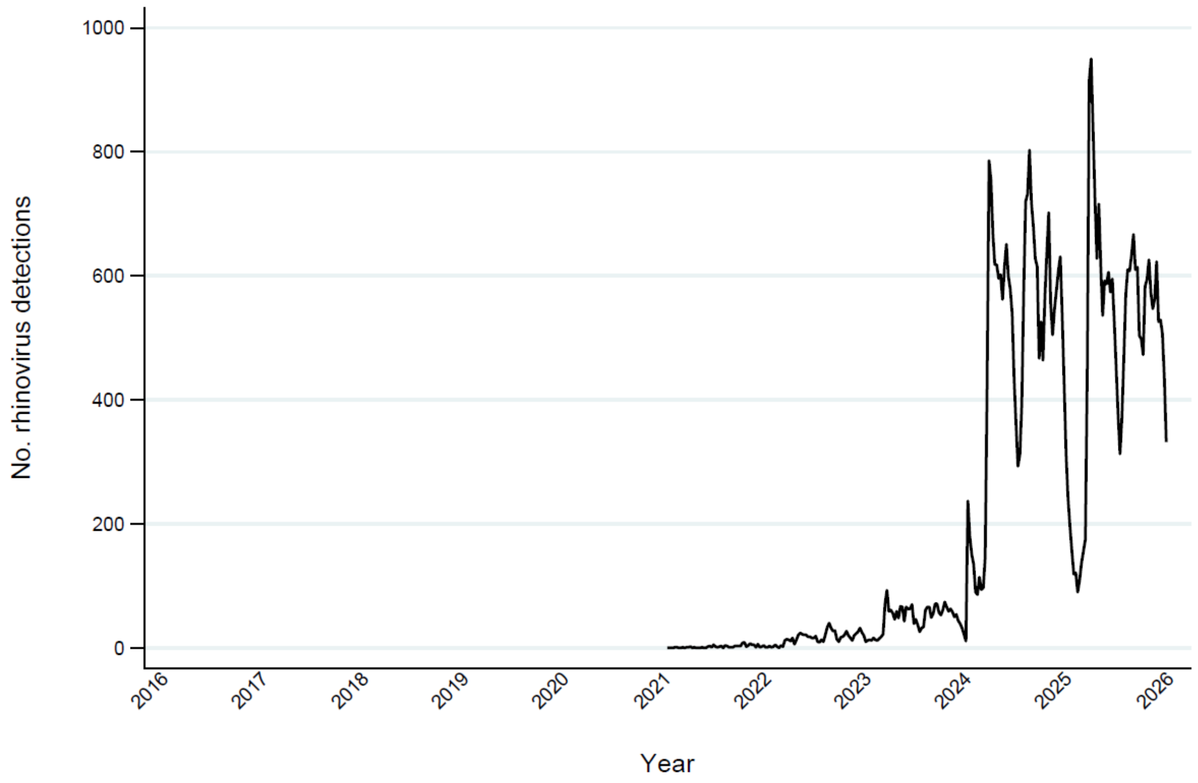

Supplementary Figure S10: Time-series of rhinovirus circulation in Colombia. WHO FluNet, 2016-2025.

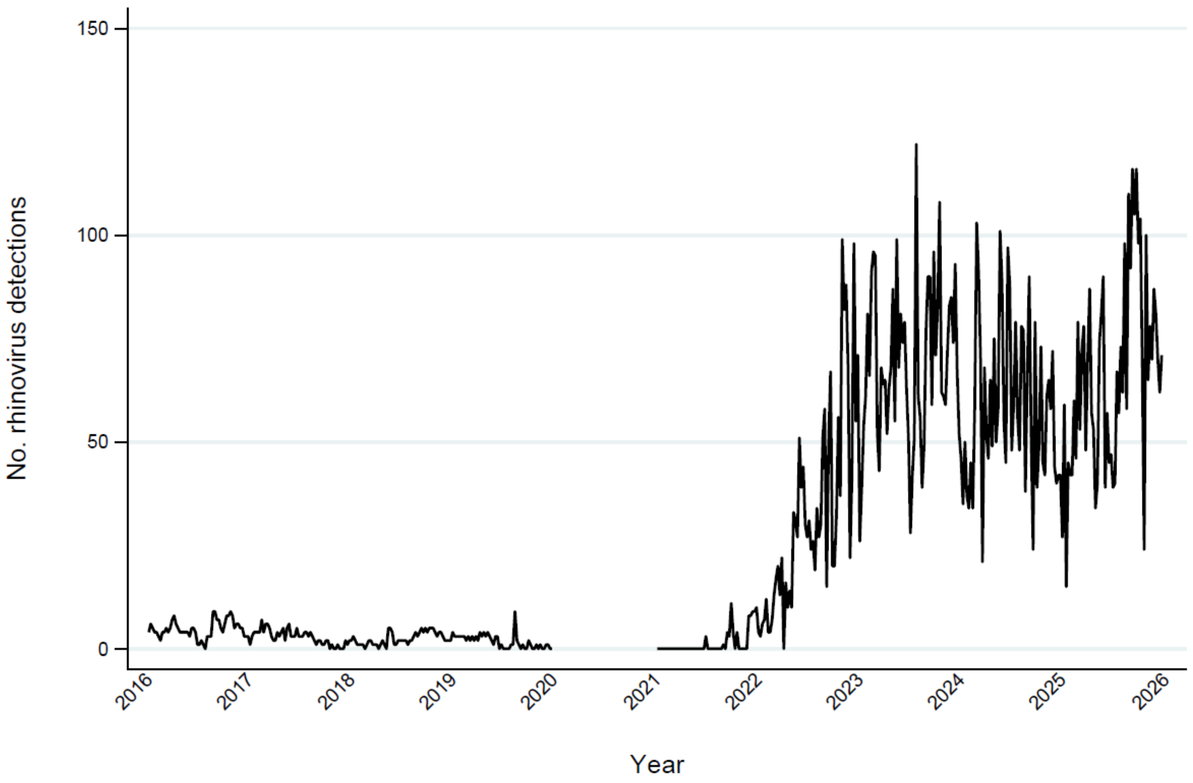

Supplementary Figure S11: Time-series of rhinovirus circulation in Costa Rica. WHO FluNet, 2016-2025.

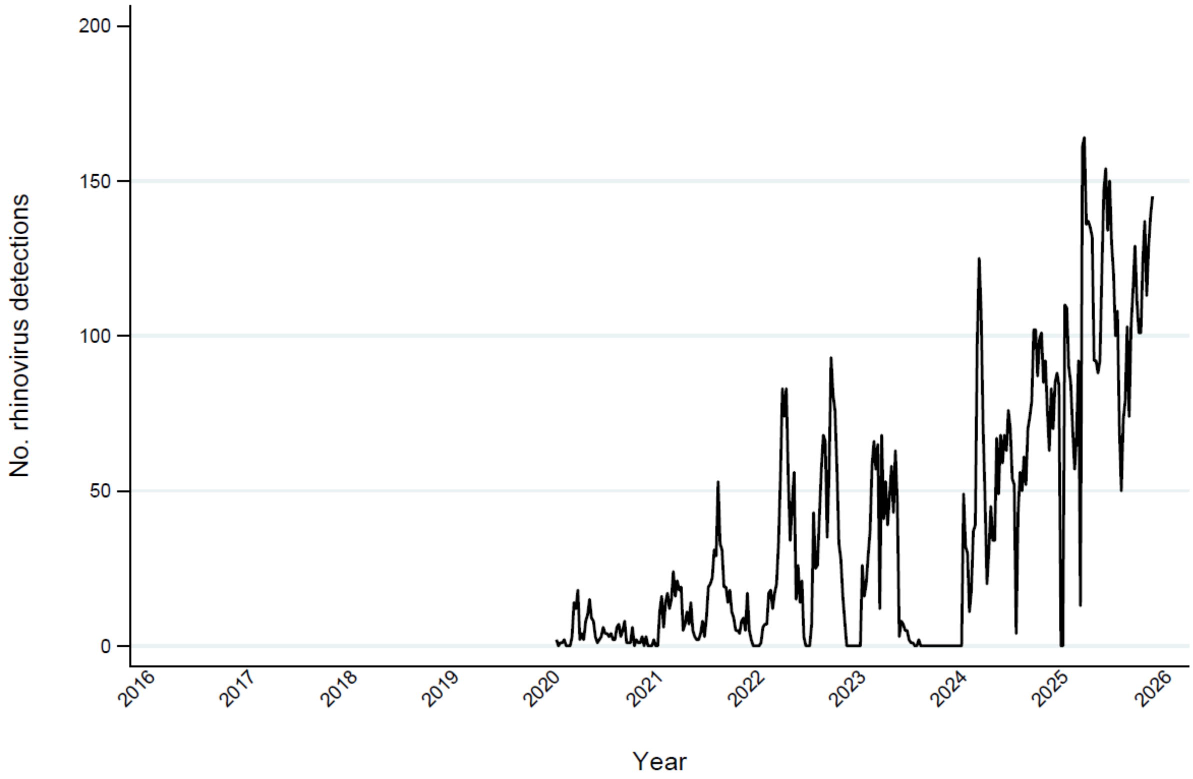

Supplementary Figure S12: Time-series of rhinovirus circulation in Cuba. WHO FluNet, 2016-2025.

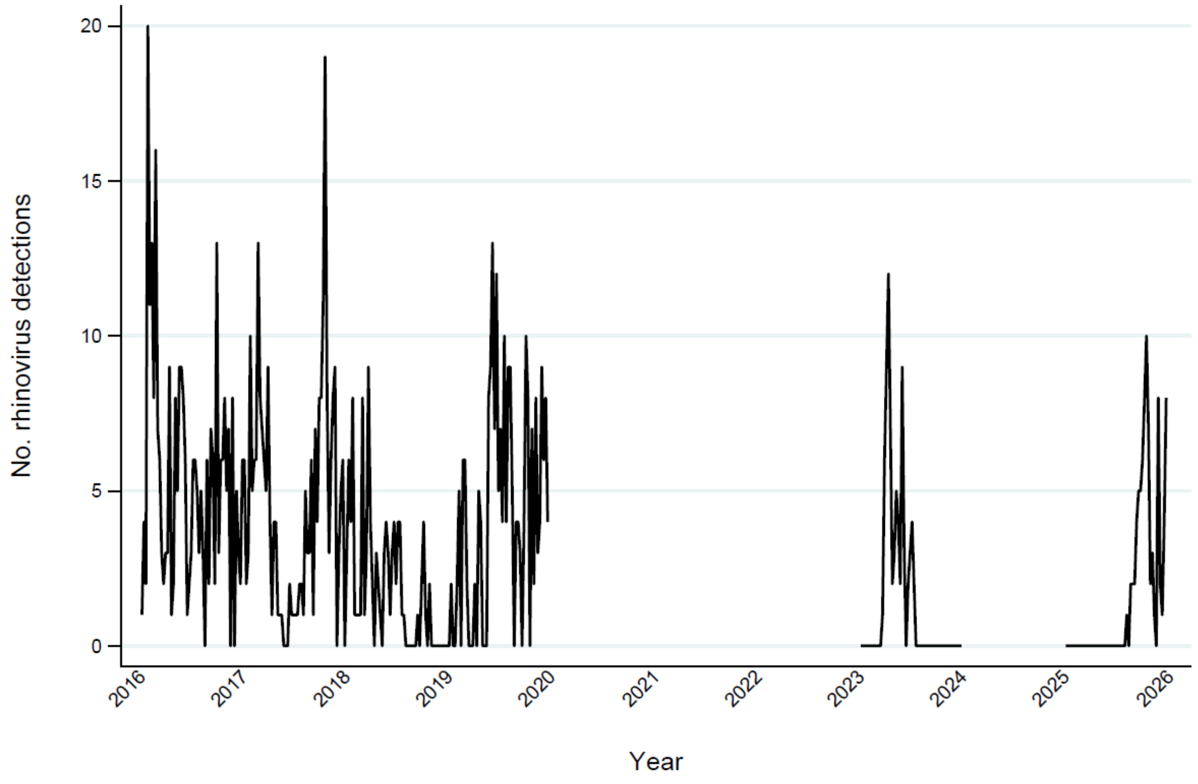

Supplementary Figure S13: Time-series of rhinovirus circulation in El Salvador. WHO FluNet, 2016-2025.

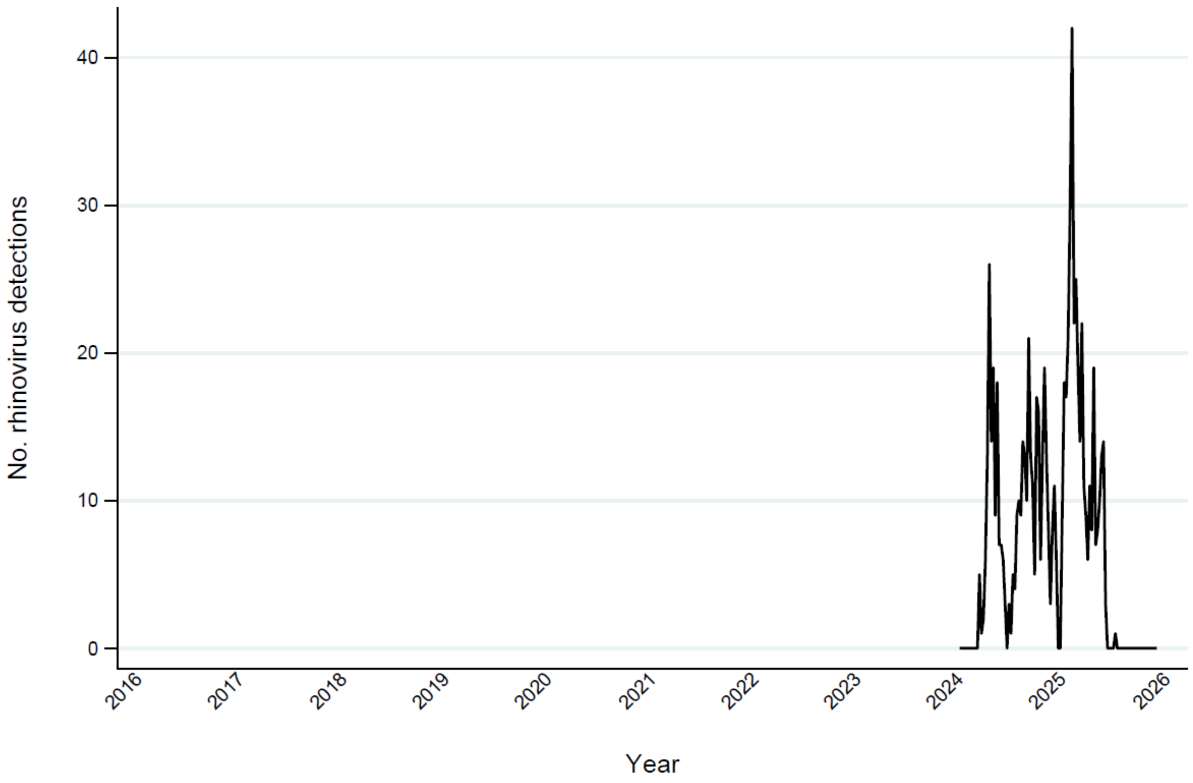

Supplementary Figure S14: Time-series of rhinovirus circulation in Guatemala. WHO FluNet, 2016-2025.

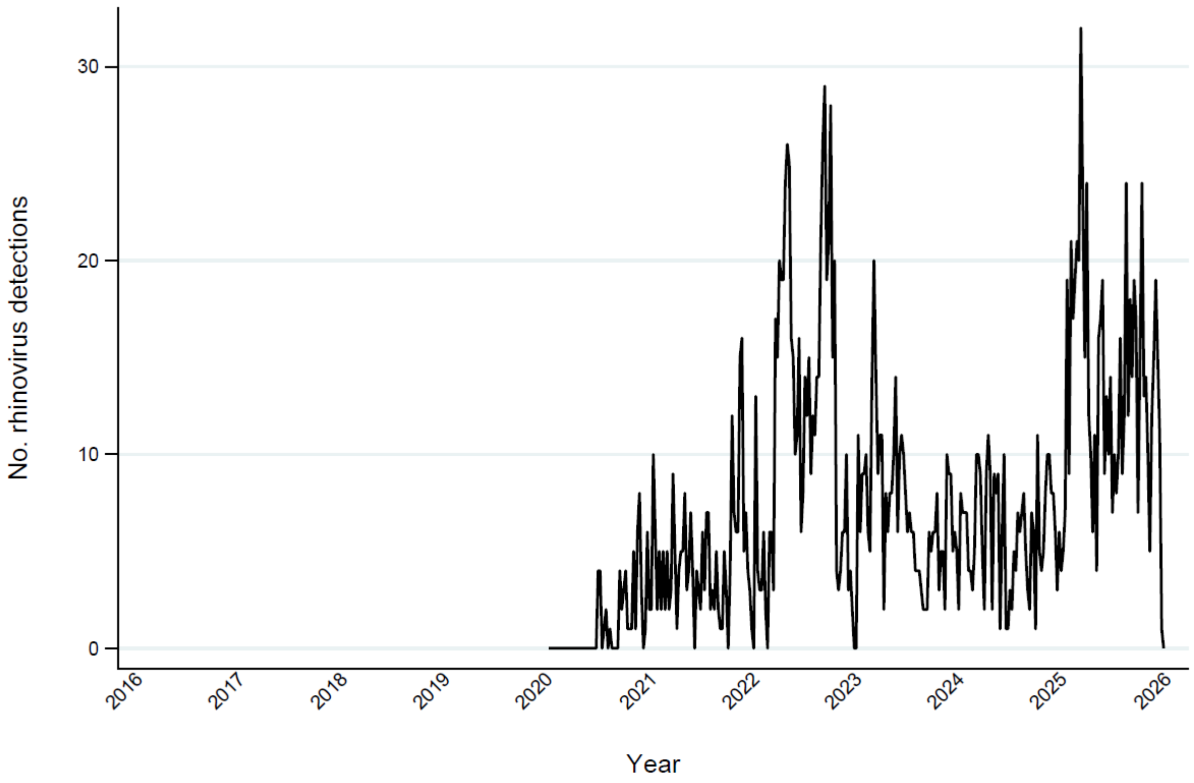

Supplementary Figure S15: Time-series of rhinovirus circulation in India. WHO FluNet, 2016-2025.

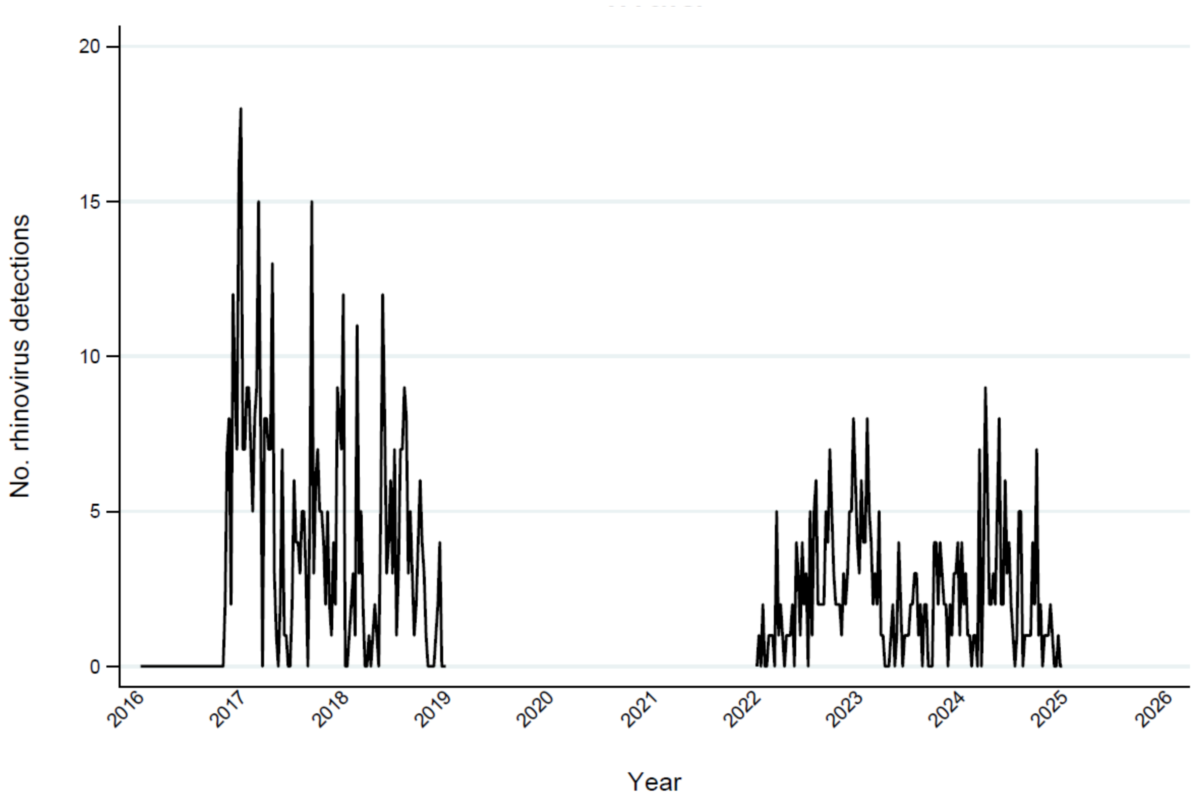

Supplementary Figure S16: Time-series of rhinovirus circulation in Japan. WHO FluNet, 2016-2025.

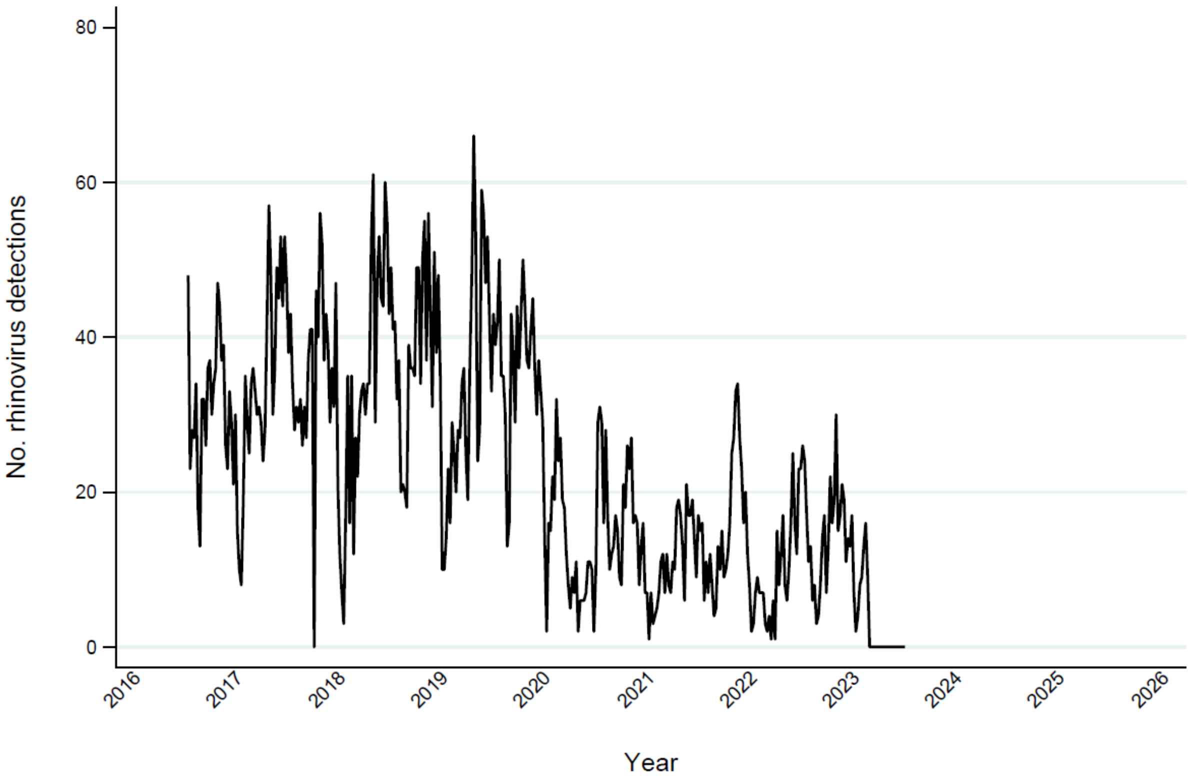

Supplementary Figure S17: Time-series of rhinovirus circulation in Kuwait. WHO FluNet, 2016-2025.

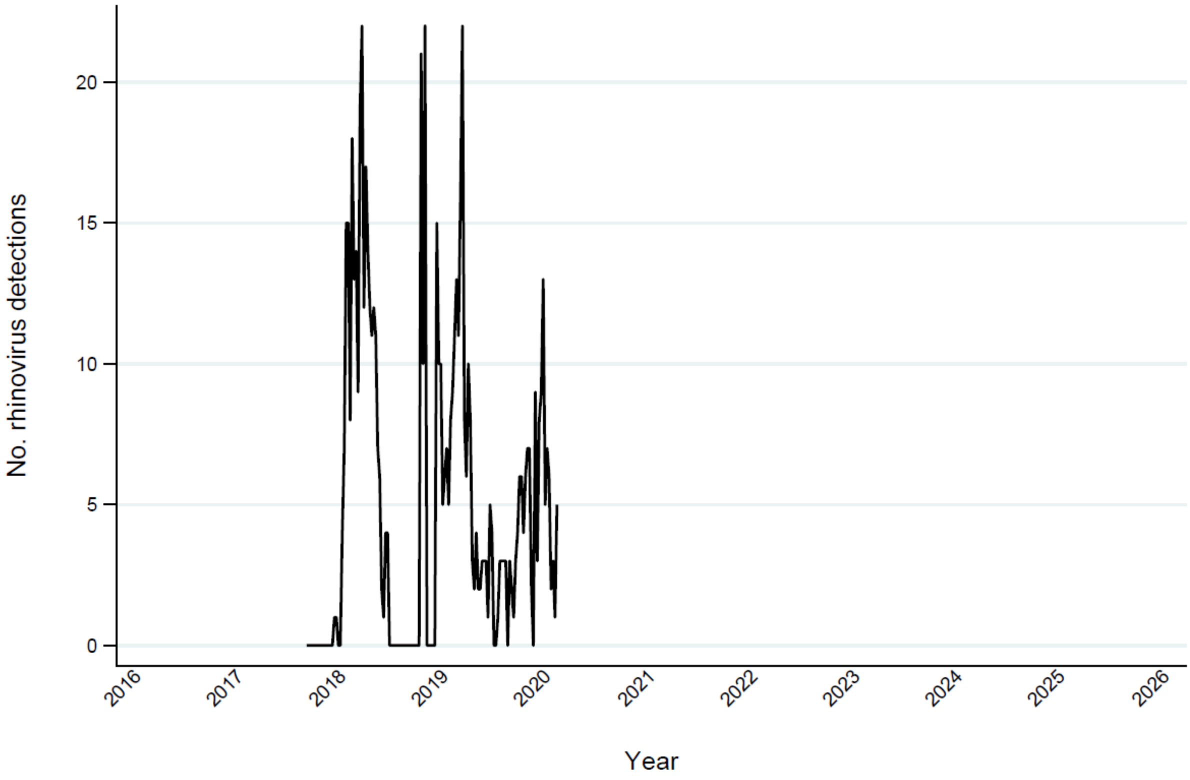

Supplementary Figure S18: Time-series of rhinovirus circulation in Mexico. WHO FluNet, 2016-2025.

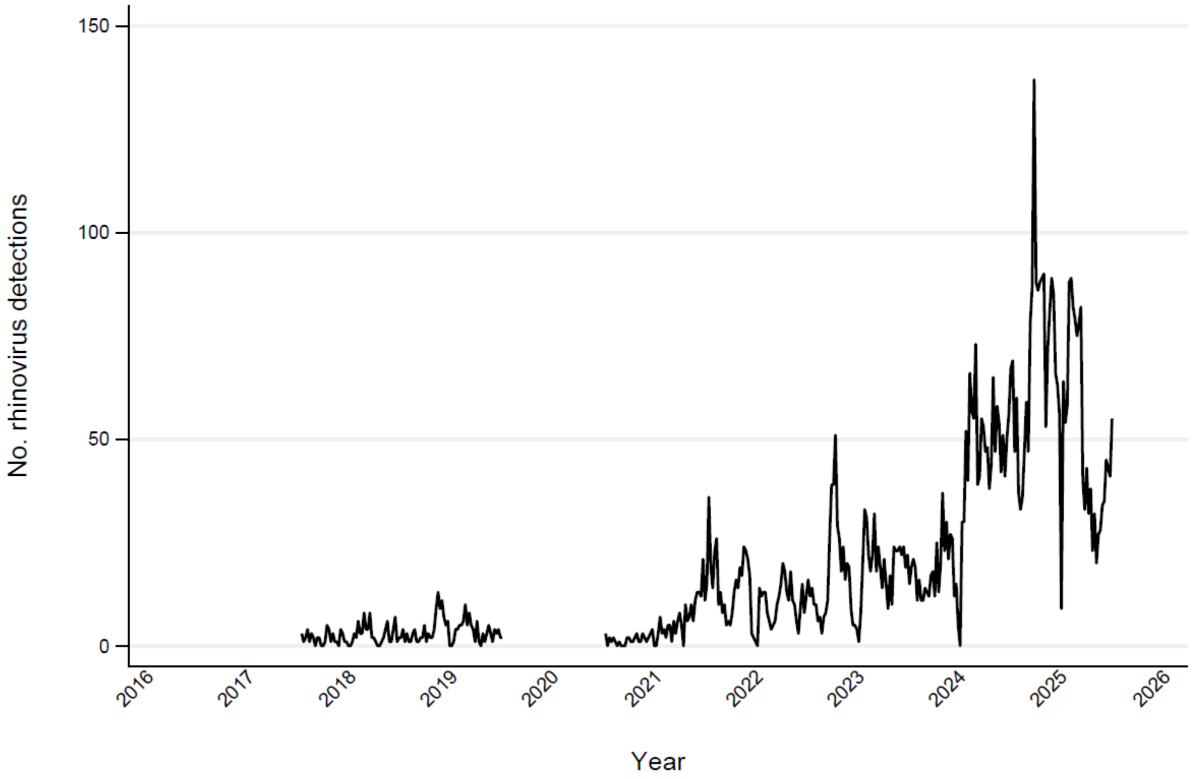

Supplementary Figure S19: Time-series of rhinovirus circulation in Mongolia. WHO FluNet, 2016-2025.

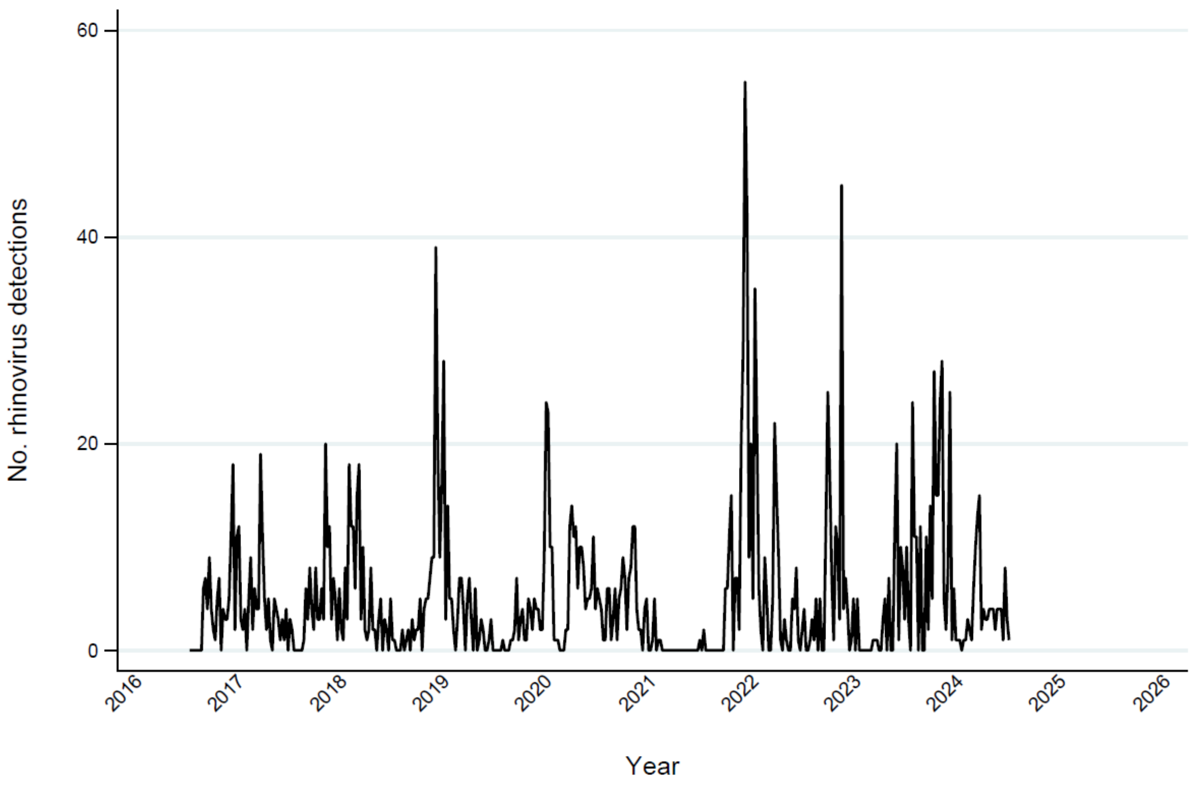

Supplementary Figure S20: Time-series of rhinovirus circulation in New Zealand. WHO FluNet, 2016-2025.

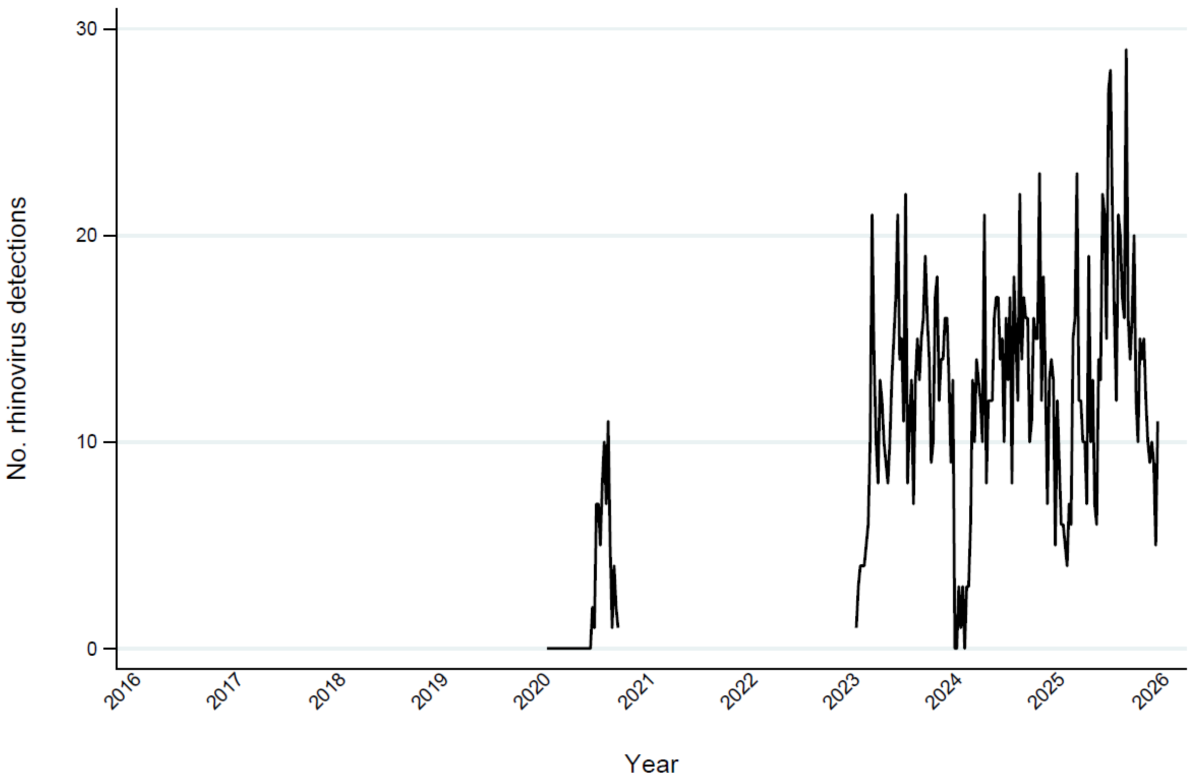

Supplementary Figure S21: Time-series of rhinovirus circulation in Nicaragua. WHO FluNet, 2016-2025.

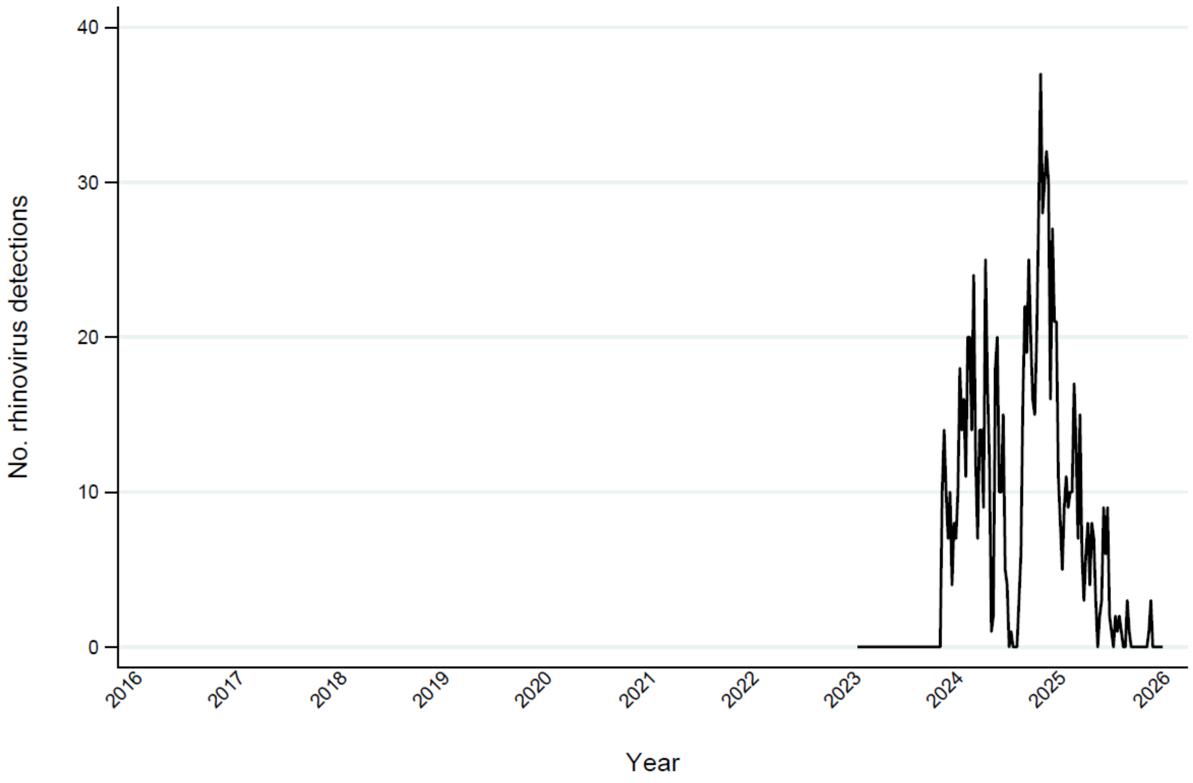

Supplementary Figure S22: Time-series of rhinovirus circulation in Oman. WHO FluNet, 2016-2025.

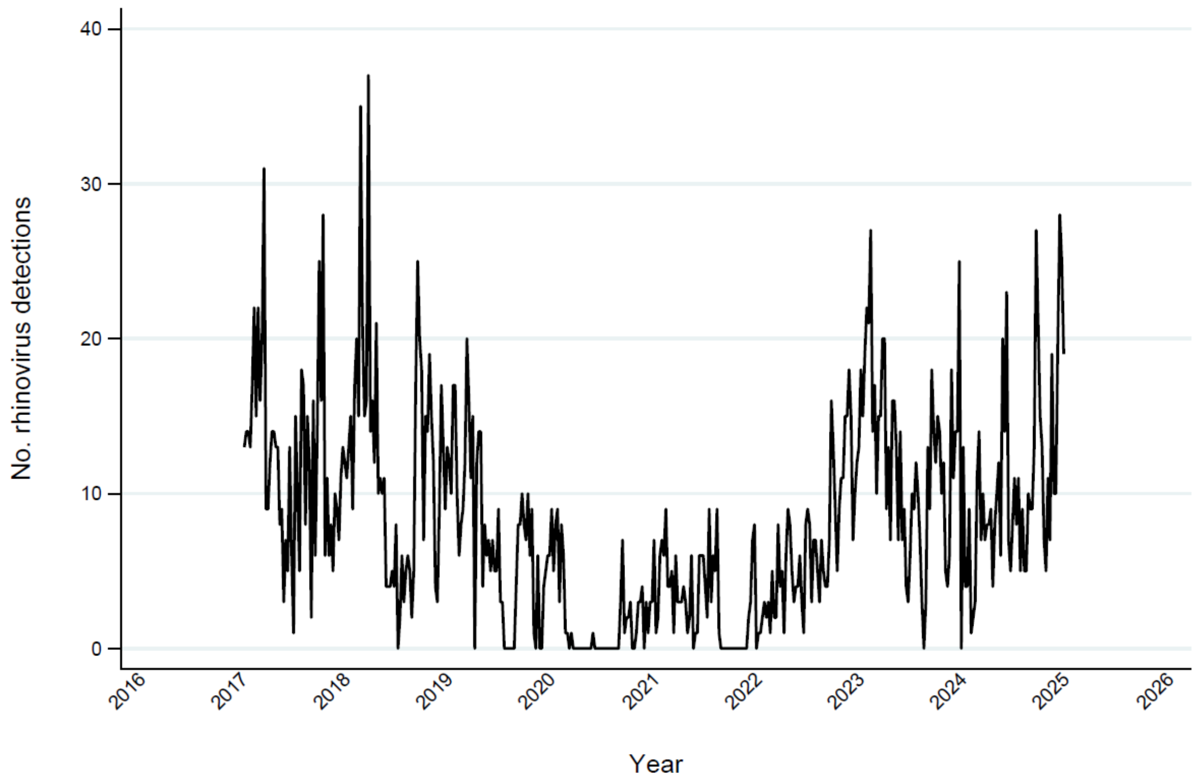

Supplementary Figure S23: Time-series of rhinovirus circulation in Panama. WHO FluNet, 2016-2025.

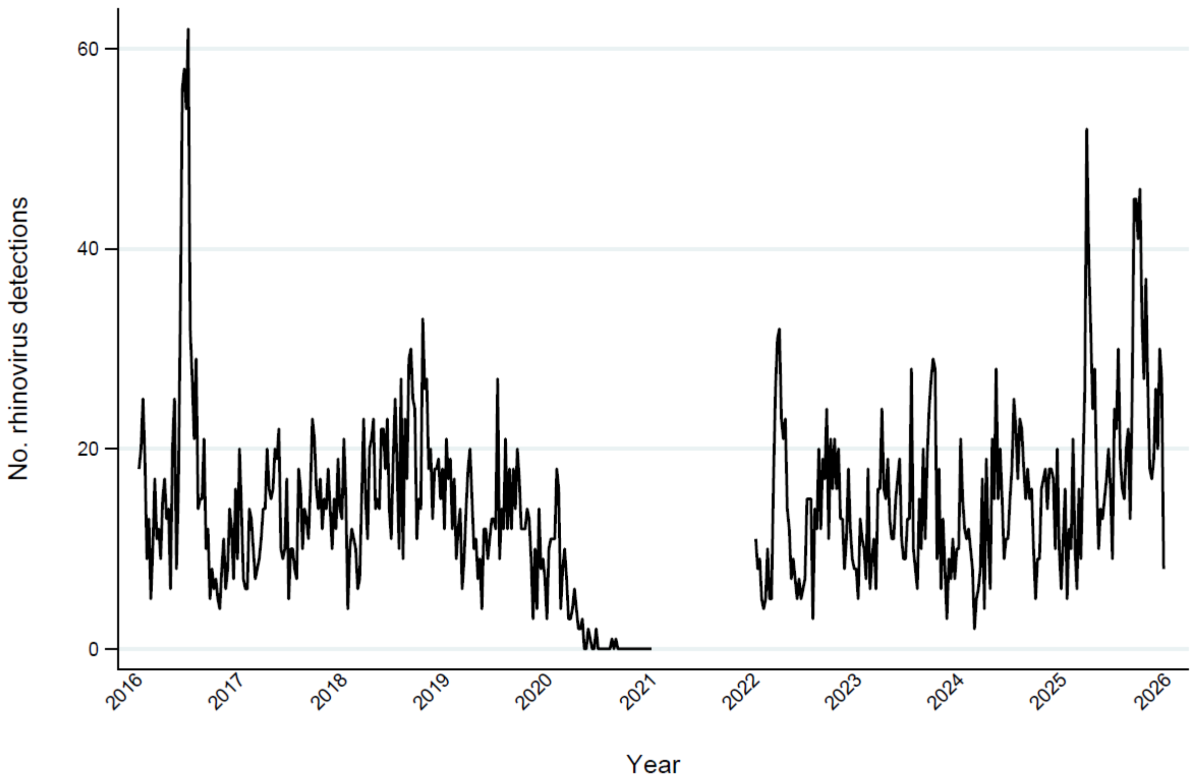

Supplementary Figure S24: Time-series of rhinovirus circulation in Paraguay. WHO FluNet, 2016-2025.

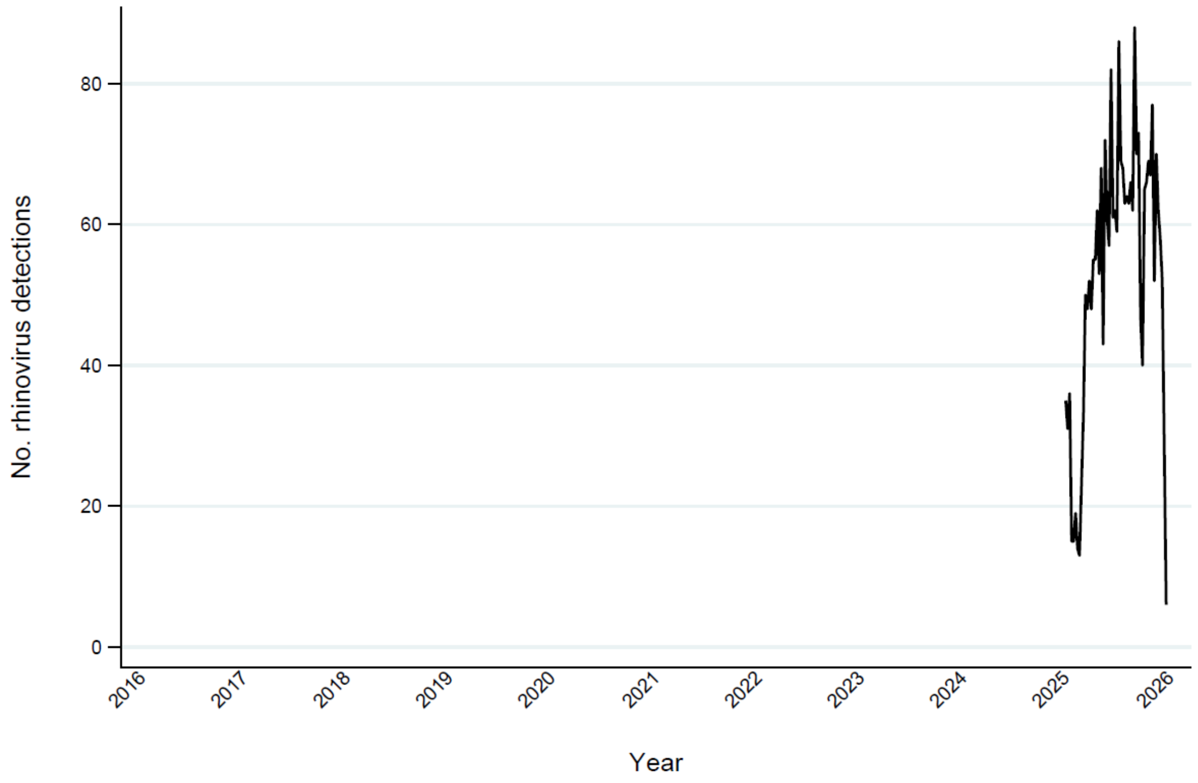

Supplementary Figure S25: Time-series of rhinovirus circulation in Peru. WHO FluNet, 2016-2025.

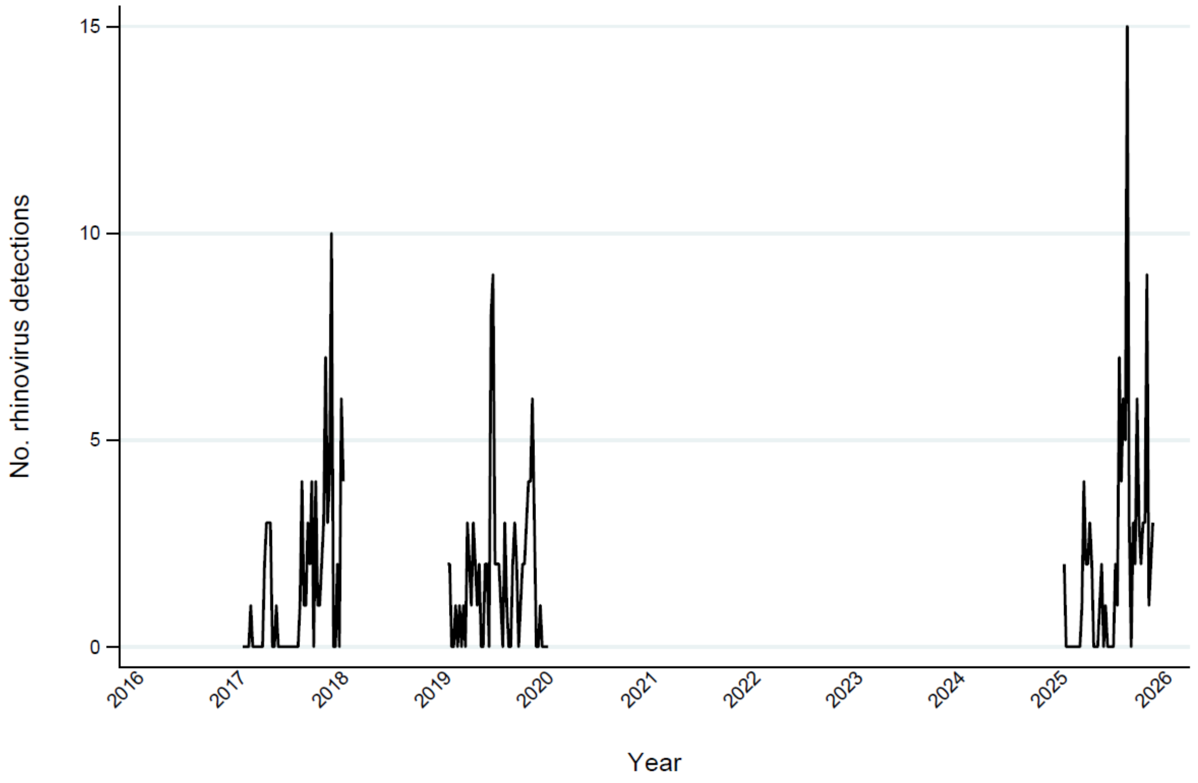

Supplementary Figure S26: Time-series of rhinovirus circulation in the Philippines. WHO FluNet, 2016-2025.

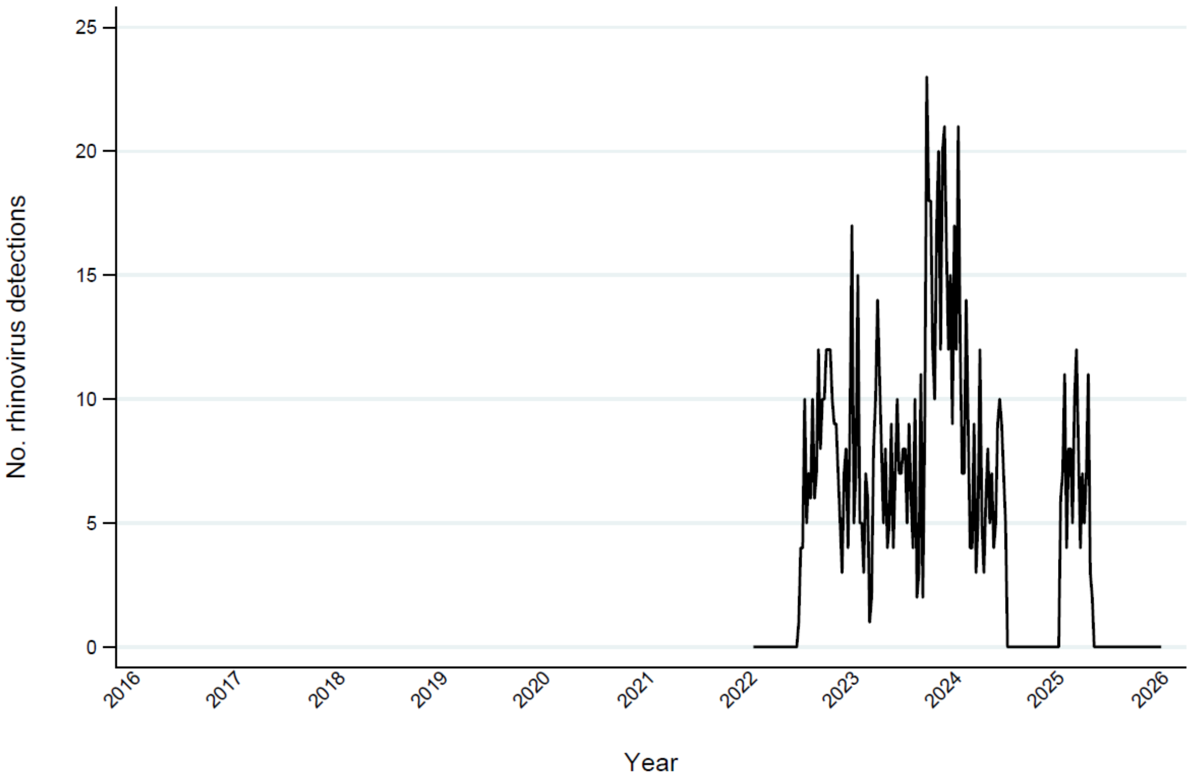

Supplementary Figure S27: Time-series of rhinovirus circulation in Qatar. WHO FluNet, 2016-2025.

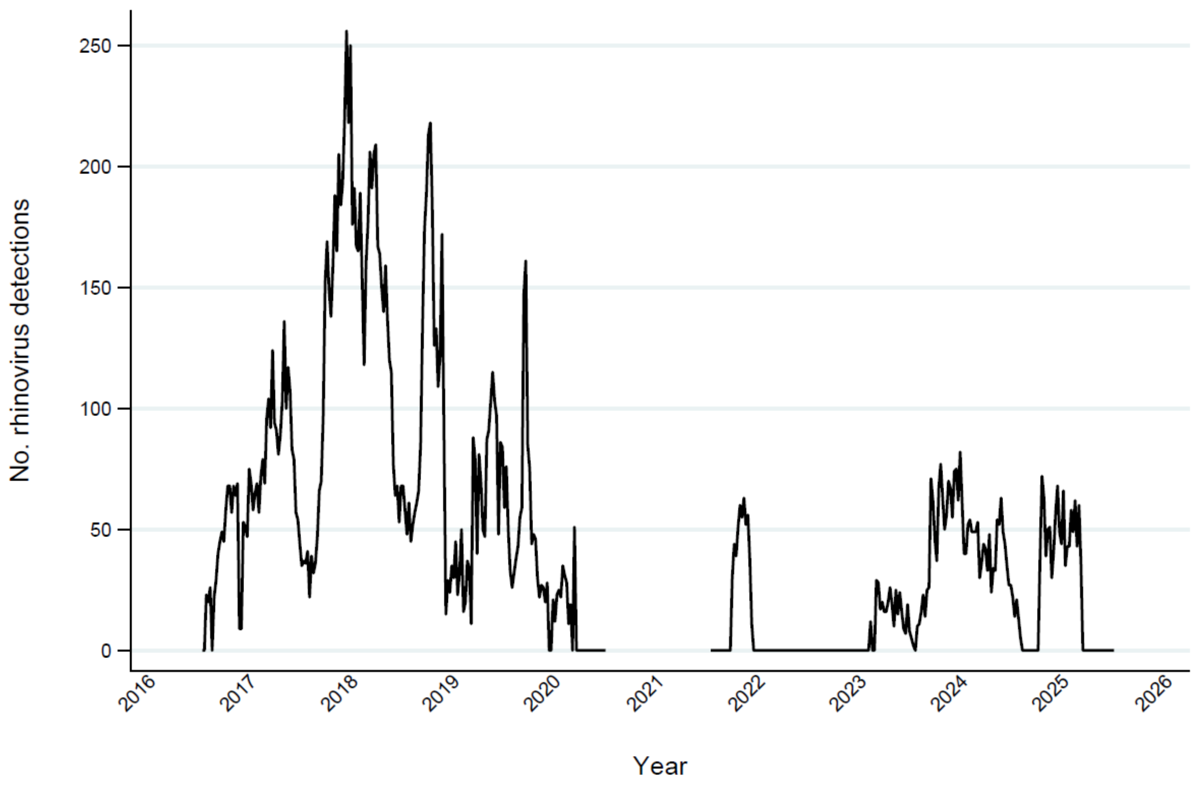

Supplementary Figure S28: Time-series of rhinovirus circulation in Senegal. WHO FluNet, 2016-2025.

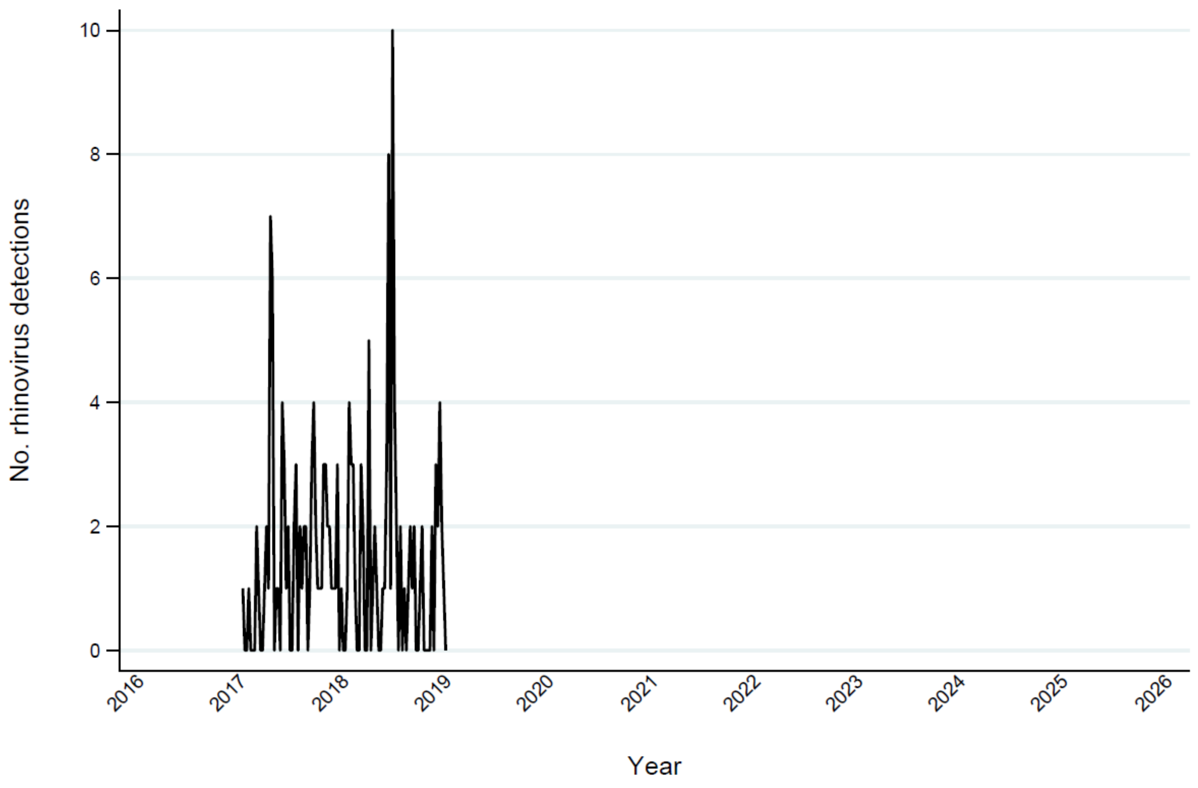

Supplementary Figure S29: Time-series of rhinovirus circulation in South Africa. WHO FluNet, 2016-2025.

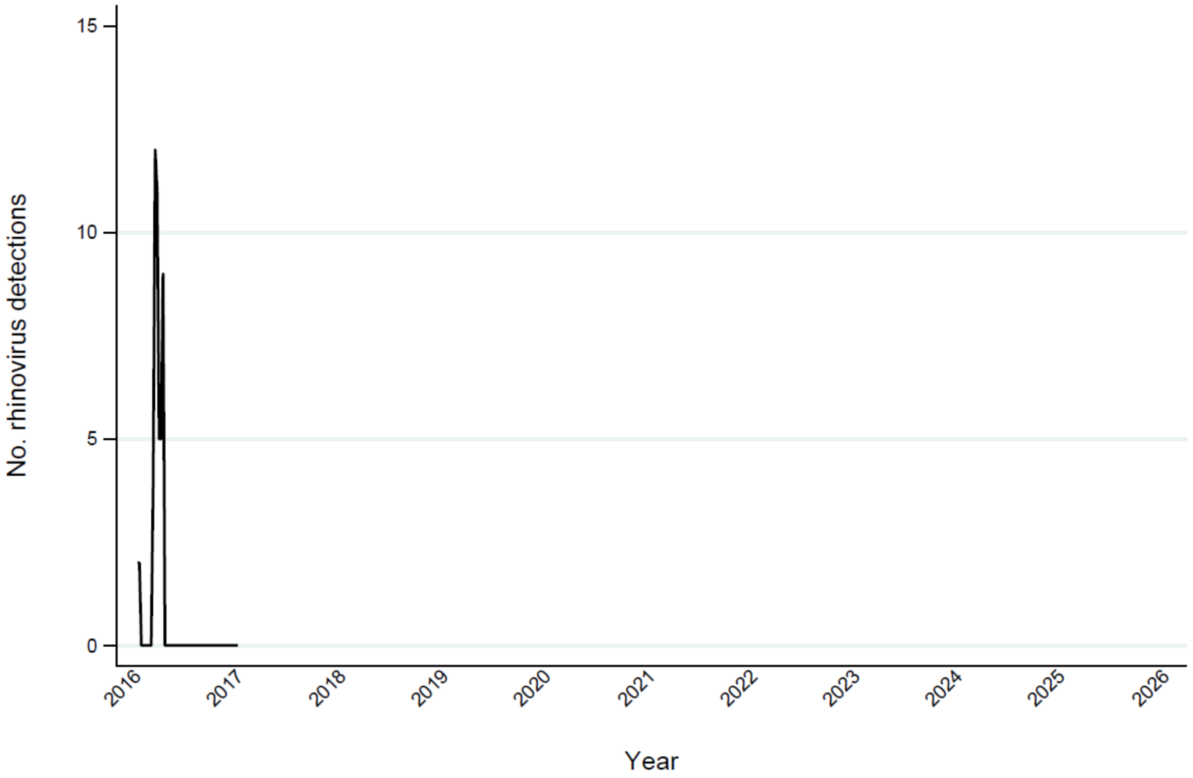

Supplementary Figure S30: Time-series of rhinovirus circulation in Thailand. WHO FluNet, 2016-2025.

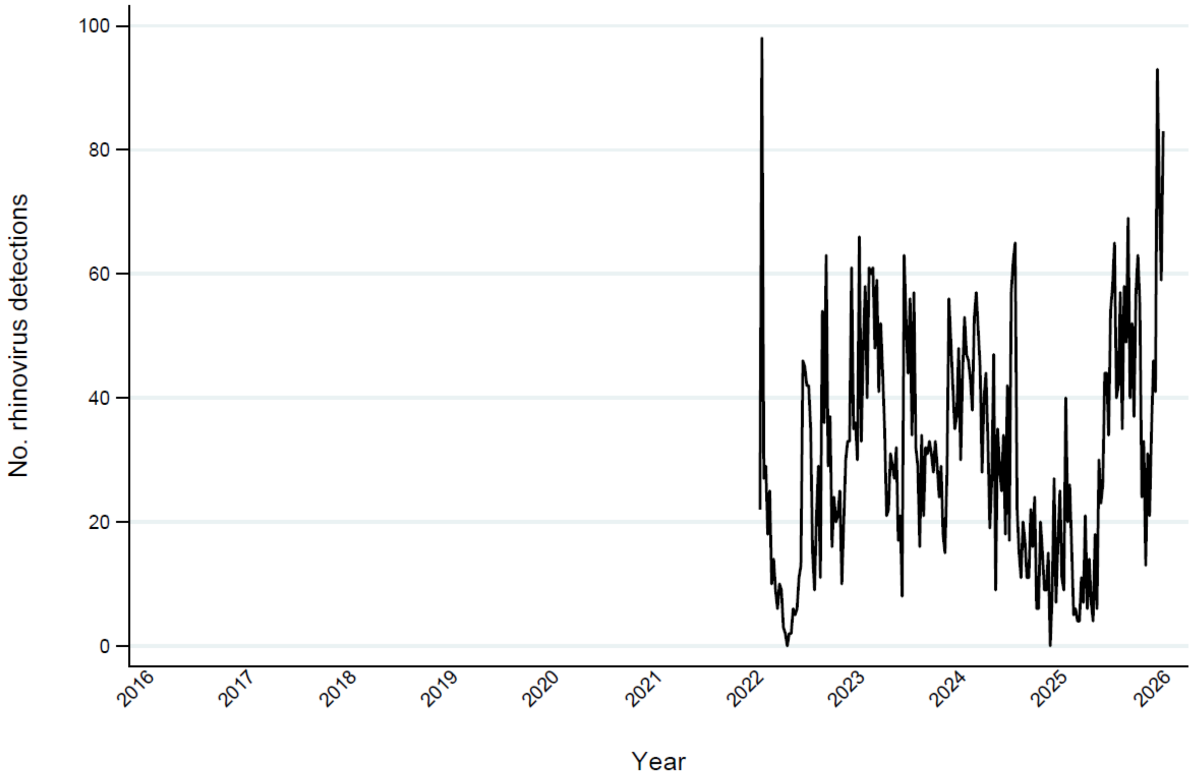

Supplementary Figure S31: Time-series of rhinovirus circulation in Tunisia. WHO FluNet, 2016-2025.

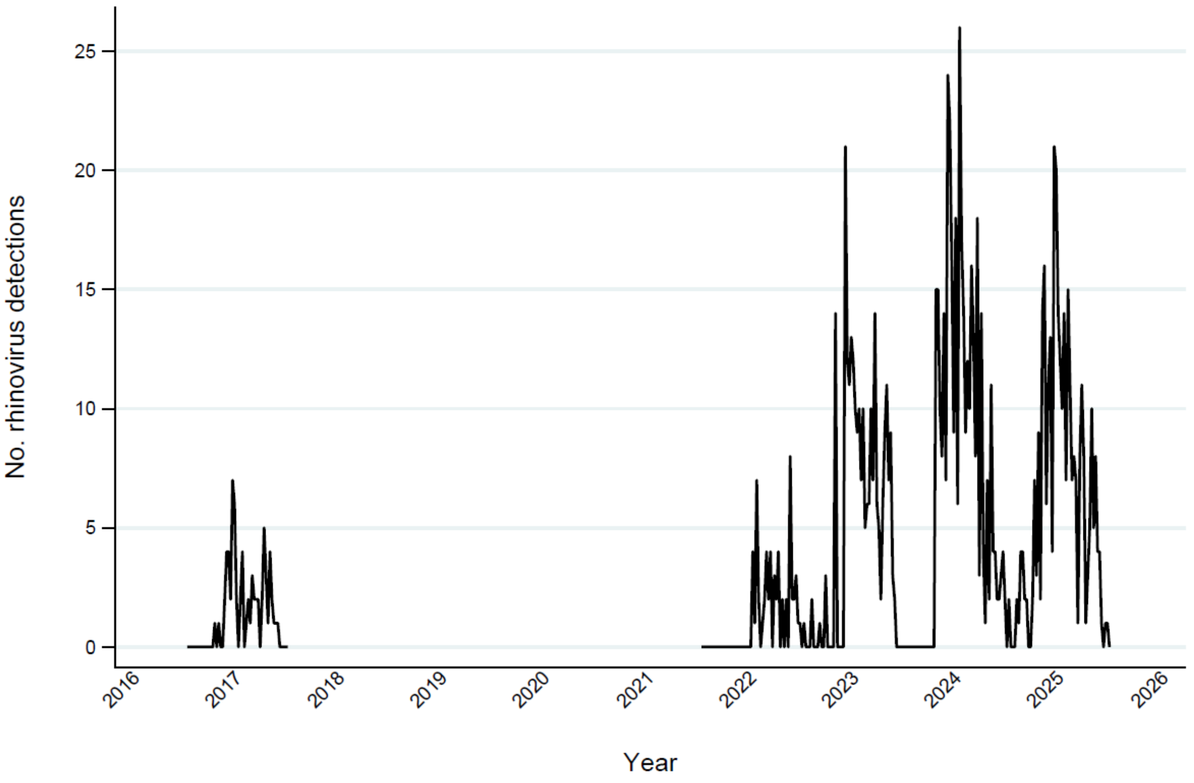

Supplementary Figure S32: Time-series of rhinovirus circulation in the United Arab Emirates. WHO FluNet, 2016-2025.

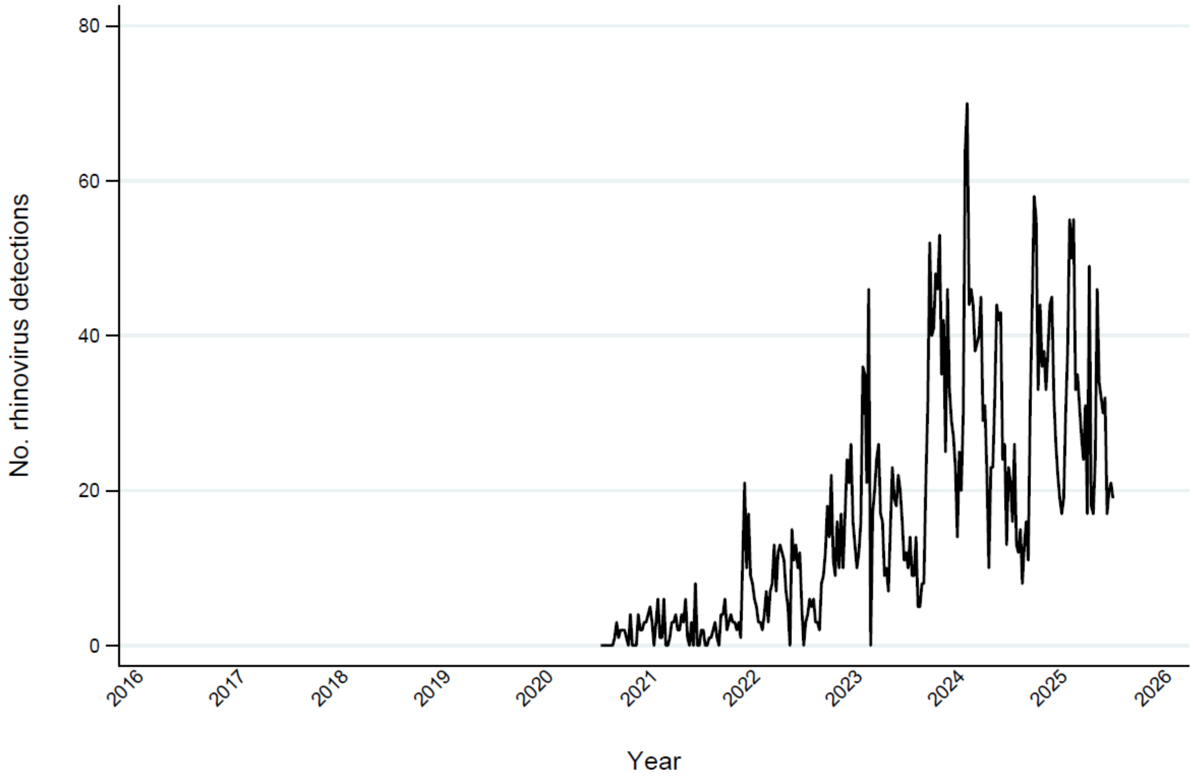

Supplementary Figure S33: Time-series of rhinovirus circulation in Uruguay. WHO FluNet, 2016-2025.

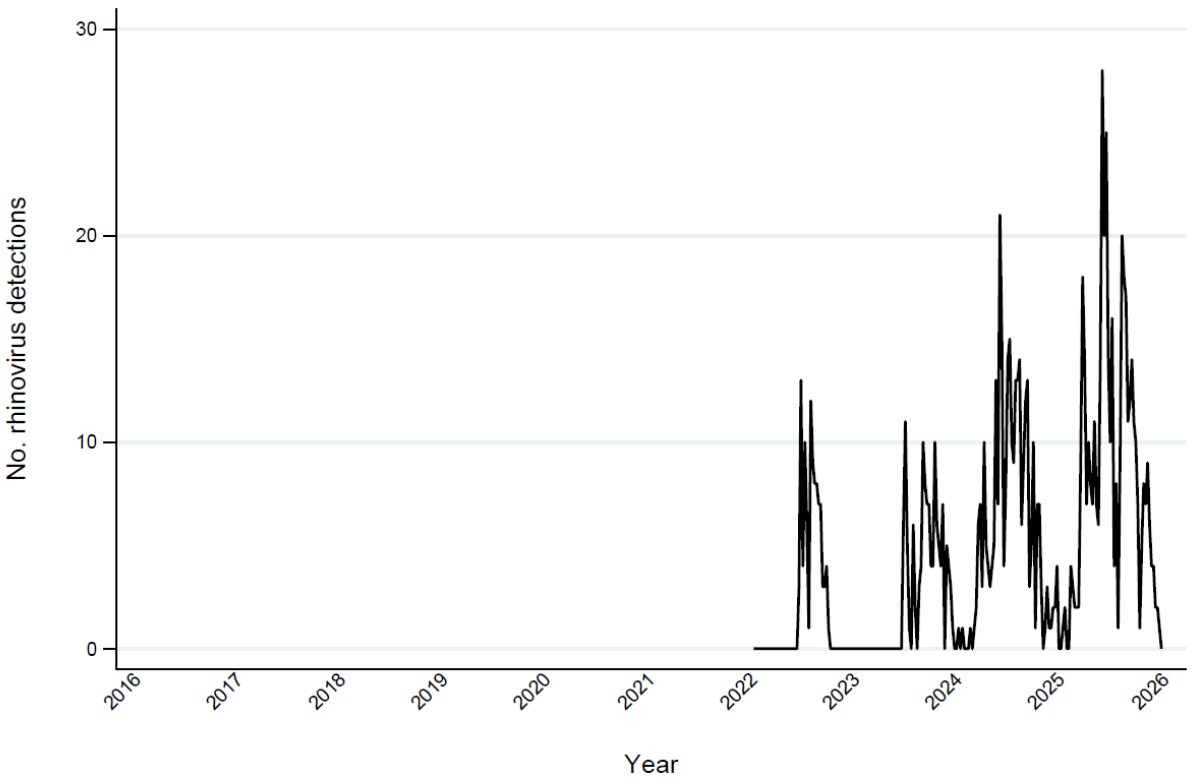

Supplementary Figure S34: Time-series of rhinovirus circulation in Viet Nam. WHO FluNet, 2016-2025.

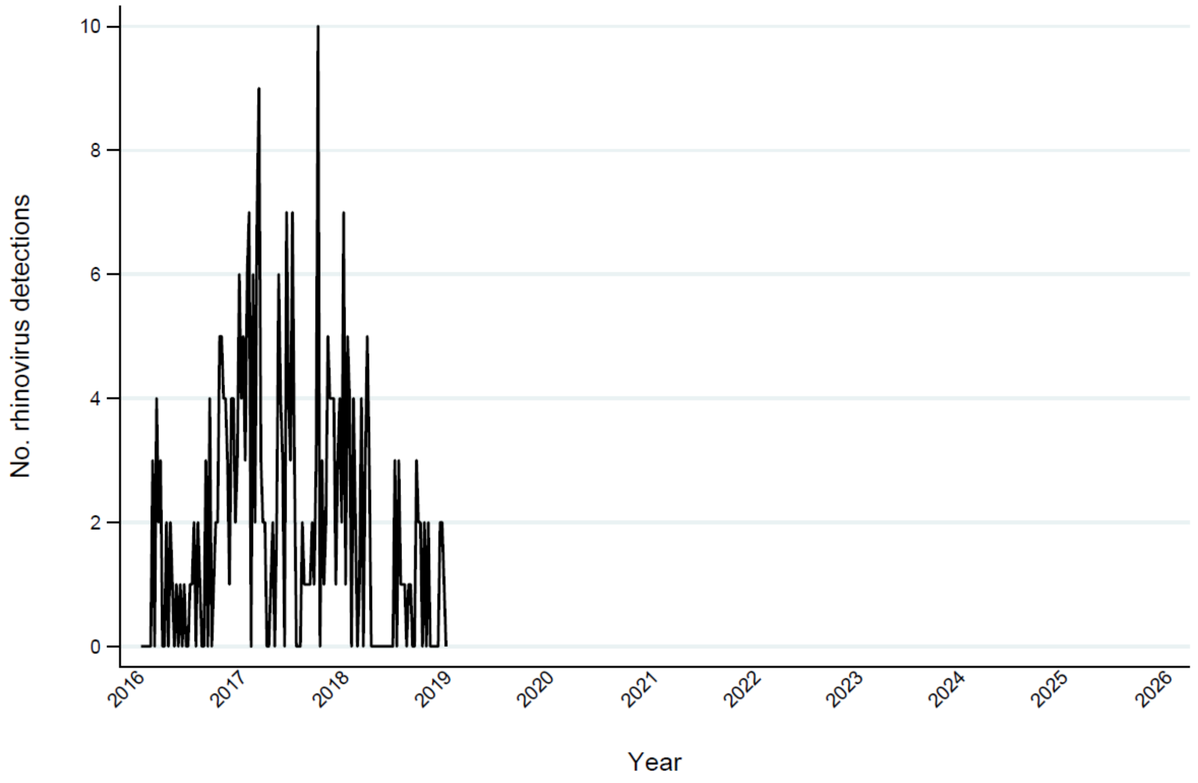

Supplement: Supplementary file 1 [file pathogens-15-00446-s001.zip › pathogens-4229028-SM-Figure S1-S34.pdf]
