# Supplementary material for: Global Patterns of Human Rhinovirus Activity and Epidemic Duration, 2016–2025: Before, During, and After the COVID-19 Pandemic
Source: Pathogens. 2026 Apr 20;15(4):446. doi: 10.3390/pathogens15040446 (PMC13119256; doi:10.3390/pathogens15040446)
Supplement: Supplementary file 1 [file pathogens-15-00446-s001.zip › Supplementary Table S1 .pdf]

Supplementary Table S1: Global circulation of rhinovirus in countries lying in the Northern hemisphere, inter-tropical belt, or Southern hemisphere. WHO FluNet, 2016-2025.

Geographical area	N of rhinovirus detections reported to Flunet	Median detections per season	N (%) seasons with 1-24 reported cases	N (%) seasons with 25-49 reported cases	N (%) seasons with ≥ 50 reported cases
Northern hemisphere	251,737	382	7 (11.7%)	2 (3.3%)	51 (85.0%)
Inter-tropical belt	69,649	89	44 (26.7%)	20 (12.1%)	101 (61.2%)
Southern hemisphere	111,013	1,924	1 (4.0%)	0 (0.0%)	24 (96.0%)
**Total**	**432,399**	**171**	**52 (20.8%)**	**22 (8.8%)**	**176 (70.4%)**
