# Supplementary material for: Global Patterns of Human Rhinovirus Activity and Epidemic Duration, 2016–2025: Before, During, and After the COVID-19 Pandemic
Source: Pathogens. 2026 Apr 20;15(4):446. doi: 10.3390/pathogens15040446 (PMC13119256; doi:10.3390/pathogens15040446)
Supplement: Supplementary file 1 [file pathogens-15-00446-s001.zip › Supplementary Table S2 .pdf]

Supplementary Table S2: Global circulation of rhinovirus in countries belonging to the different WHO regions. WHO FluNet, 2016-2025.

WHO region	N of rhinovirus detections reported to Flunet	Median detections per season	N (%) seasons with 1-24 reported cases	N (%) seasons with 25-49 reported cases	N (%) seasons with ≥ 50 reported cases
African Region (AFR)	1,299	30	8 (40.0%)	3 (15.0%)	9 (45.0%)
Region of the Americas (AMR)	324,215	132	28 (22.0%)	12 (9.5%)	87 (68.5%)
Eastern Mediterranean (EMR)	30,389	331	7 (18.4%)	1 (2.6%)	30 (71.0%)
European Region (EUR)	0	-	-	-	-
South-East Asia (SEAR)	7,521	113	4 (23.5%)	3 (17.7%)	10 (58.8%)
Western Pacific (WPR)	68,975	365	5 (10.4%)	3 (6.3%)	40 (83.3%)
**Total**	**432,399**	**171**	**52 (20.8%)**	**22 (8.8%)**	**176 (70.4%)**
