# Supplementary material for: Global Patterns of Human Rhinovirus Activity and Epidemic Duration, 2016–2025: Before, During, and After the COVID-19 Pandemic
Source: Pathogens. 2026 Apr 20;15(4):446. doi: 10.3390/pathogens15040446 (PMC13119256; doi:10.3390/pathogens15040446)
Supplement: Supplementary file 1 [file pathogens-15-00446-s001.zip › Supplementary Table S3 .pdf]

Supplementary Table S3: Global circulation of rhinovirus by season. WHO FluNet, 2016-2025.

Season	N of rhinovirus detections reported to Flunet	Median detections per season	N (%) seasons with 1-24 reported cases	N (%) seasons with 25-49 reported cases	N (%) seasons with ≥ 50 reported cases
2016	25,698	81	3 (15.8%)	4 (21.1%)	12 (63.2%)
2017	29,477	145	6 (24.0%)	2 (8.0%)	17 (68.0%)
2018	25,765	98	7 (25.9%)	3 (11.1%)	17 (63.0%)
2019	10,461	70	8 (33.3%)	3 (12.5%)	13 (54.2%)
2020	31,080	70	8 (36.4%)	1 (4.5%)	13 (51.1%)
2021	34,889	282	2 (10.5%)	2 (10.5%)	15 (79.0%)
2022	45,462	250	5 (17.9%)	0 (0.0%)	23 (82.1%)
2023	62,844	379	6 (20.0%)	0 (0.0%)	24 (80.0%)
2024	110,650	321	4 (12.9%)	5 (16.1%)	22 (71.0%)
2025	56,073	374	3 (12.0%)	2 (8.0%)	20 (80.0%)
**Total**	**432,399**	**171**	**52 (20.8%)**	**22 (8.8%)**	**176 (70.4%)**
